# Battling dengue in a warming world: How climate and urbanization shape transmission in low- and middle-income countries (a rapid review)

**DOI:** 10.1371/journal.pntd.0013758

**Published:** 2026-07-20

**Authors:** Nicole Saad, Jiaqin Wu, Myha Hill, Scott Dorris, Jingyi Liu, Finn Wernet, Yu-Hsiang Wu, Jasnoor Kaur Anand, Abrahim Sawez, Weijun Yu

**Affiliations:** 1 College of Arts and Sciences, Georgetown University, Washington, DC, United States of America; 2 Center for Global Health Practice and Impact, Georgetown University Medical Center, Washington, DC, United States of America; 3 School of Health, Georgetown University, Washington, DC, United States of America; 4 Dahlgren Memorial Library, Georgetown University, Washington, DC, United States of America; 5 Walsh School of Foreign Service, Georgetown University, Washington, DC, United States of America; 6 Charité - Universitätsmedizin Berlin, Berlin, Germany; Public Health Agency of Canada, CANADA

## Abstract

**Background:**

Dengue fever is a globally prevalent vector-borne disease, with low- and middle-income countries (LMICs) experiencing a disproportionate burden. Transmission patterns are increasingly influenced by rising global temperatures and rapid urbanization, yet no recent review has synthesized how these environmental factors are reshaping dengue dynamics in LMICs.

**Methods:**

We conducted a rapid systematic review following a registered protocol (PROSPERO: CRD42025635982). Six databases (MEDLINE, Embase, Global Health, SciELO, Global Index Medicus and Web of Science) were searched for peer-reviewed studies published between January 2020 and November 2024. Studies were screened for climate or urbanization factors affecting dengue transmission in LMICs. Study quality was assessed using the Mixed Methods Appraisal Tool for empirical studies and a structured assessment for modeling studies. Findings were synthesized narratively.

**Results:**

Of the 65 included studies, 65% reported a positive association between rising temperatures and increased dengue transmission, often attributed to intensified mosquito activity and longer transmission seasons. Urbanization was assessed in 35% of studies, with unplanned growth, informal settlements, and inadequate infrastructure frequently associated with increased risk. Several studies reported geographic expansion into highland and peri-urban areas, as well as seasonal shifts, including earlier onset and prolonged transmission periods. Urban heat islands and land-use change were commonly associated with localized transmission hotspots. Government responses were documented in 29% of studies, though many were hindered by fragmented surveillance systems, limited cross-sector coordination, or insufficient integration of climate data into public health planning.

**Conclusion:**

Climate change and urbanization are reshaping the spatial and temporal dynamics of dengue in LMICs. Adaptive responses require strengthened surveillance and climate-informed planning in resilient urban infrastructure to reduce future transmission risks.

## Author summary

Dengue fever is spreading in many low- and middle-income countries, and this review helps explain how climate and urbanization are contributing to that change. We reviewed recent studies published between 2020 and 2024 to understand how rising temperatures, rainfall patterns, humidity, and urban development are affecting where and when dengue occurs. We found that warmer temperatures were often linked with increased dengue transmission, partly because mosquitoes can become more active and the virus can spread more efficiently under certain temperature conditions. We also found that unplanned urban growth, crowded housing, poor drainage, and limited access to clean water can create more places for mosquitoes to breed. Several studies showed that dengue is appearing in new areas, including higher-altitude and peri-urban regions, and that transmission seasons may be starting earlier or lasting longer. Our review also identified early implementation of government-led early warning systems and risk mapping tools; however, many response efforts remain constrained by fragmented systems, limited intersectoral coordination, and insufficient resources. These findings show the need for climate-informed public health planning and resilient urban infrastructure to reduce future dengue transmission risk.

## Introduction

Dengue fever is one of the fastest-growing vector-borne diseases worldwide, driven largely by the global spread of *Aedes aegypti* mosquitoes and increasingly favorable environmental conditions across low- and middle-income countries (LMICs) [1]. Climate variability plays a critical role in shaping dengue transmission dynamics by influencing both mosquito breeding and viral replication. Warmer ambient temperatures can accelerate the development of *Aedes aegypt*i mosquitoes, increase biting frequency, and shorten the viral incubation period within the vector, thereby enhancing transmission potential. However, these effects are nonlinear, as both low and very high temperatures can limit mosquito survival and dengue transmission. As global temperatures and rainfall intensity continue to vary, these climate-driven mechanisms may contribute to expanded transmission seasons and increased dengue risk in many regions, while reducing suitability in others that become too warm for efficient transmission. In Central America, dengue incidence increases during wetter La Niña periods and declines during drier El Niño conditions, making the Oceanic Niño Index a useful predictor of case counts [2]. In West Africa, dengue outbreaks have been associated with specific temperature and humidity thresholds [3]. Integrating climate data into early warning systems is warranted, especially as the health and economic burden of dengue continues to rise in LMICs [4].

Efforts to control dengue vary across LMICs but are often challenged by resource constraints, fragmented surveillance, and limited public awareness. In Cambodia, although an early warning system exists, it remains disconnected from hospital infrastructure and clinical workflows [5,6]. In Papua New Guinea, mobile syndromic reporting has improved outbreak detection, but laboratory confirmation remains limited by staffing and equipment shortages [7]. Similar systemic limitations are reported in India and Yemen, where underreporting, delayed responses, and lack of community engagement hinder prevention [8,9].

Some LMICs have introduced targeted programs to strengthen dengue prevention. In the Philippines, for example, national campaigns such as the “4 o’clock habit” and “5S strategy” combine vector control with public awareness and early care-seeking behaviors [10]. Other initiatives include Sri Lanka’s 24-hour clinician helpline and India’s efforts to regulate diagnostic costs [11,12]. Nevertheless, many of these interventions remain reactive, or constrained by financial instability. In African countries where dengue is endemic, public health infrastructure is further weakened by the combined effects of poverty, limited diagnostic access, and vector breeding [13]. In such settings, dengue diagnosis often relies primarily on clinical presentation and epidemiological context due to restricted laboratory resources.

In Latin America, dengue ranks among the top neglected tropical diseases in terms of disability-adjusted life years, yet many countries have struggled to quantify its full impact or implement long-term prevention programs [14]. Transmission tends to peak during wet seasons, but responses are often inconsistent, with limited emphasis on preventive behaviors and vector control [15]. Vulnerability varies across populations, with higher infection risk observed among individuals aged 15 years and older and among residents of central, densely built-up urban areas compared with children and those living in peripheral areas [16]. Emerging co-infections with Zika and chikungunya further complicate the disease landscape and call for coordinated, climate-informed health system responses [17].

Although many LMICs routinely report dengue cases, no recent review has systematically synthesized the latest findings on how climate change and urbanization are influencing dengue dynamics in these settings. In this rapid systematic review, we address the following research question: How do climate variables and urbanization affect dengue fever transmission, including its seasonality, geographic spread, and localized risk patterns, in LMICs, and how are local governments responding to these challenges?”

## Methods

### Study design

We developed a protocol for this rapid systematic review. On January 19, 2025, our protocol was successfully registered on the International Prospective Register of Systematic Review (PROSPERO) with the registration number CRD42025635982 [18]. This study does not involve human subjects, thus ethical review and approval were formally waived by Georgetown University’s Institutional Review Board committee (IRB ID: STUDY00008665).

### Eligibility criteria

To ensure a focused analysis in our rapid review, we developed eligibility criteria outlined in Table 1. We included peer-reviewed articles written in English published between January 1st, 2020 and November 30th, 2024. Our focus was on dengue fever in relation to climate variables (including temperature, rainfall, and humidity) and urbanization in LMICs that were determined by the World Bank and continuous dengue risk by CDC databases.

**Table 1:** Eligibility Criteria.

Criterion	Inclusion Criteria	Exclusion Criteria
Population	Studies focusing on low- and middle-income countries (LMICs) that experience frequent or continuous risk of dengue fever transmission.	Studies conducted in high-income countries or regions with low or sporadic risk of dengue transmission.
Focus Area	Research and government reports addressing the effects of climate variables (including temperature, rainfall, and humidity) and urbanization on dengue fever transmission.	Studies that do not examine the impacts of climate variables or urbanization on dengue transmission or those with a primary focus on other diseases.
Government Response	Reports and studies detailing local government responses, including policies, interventions, or strategies aimed at mitigating the effects of climate and urbanization on dengue transmission.	Studies that do not focus on government response or that examine unrelated public health responses.
Language	Documents published in English.	Non-English publications unless English translation is available.
Publication Date	Studies and reports published between January 1, 2020, and November 30, 2024.	Publications before January 1, 2020, or after November 30, 2024.
Study Design and Type	Peer-reviewed journal articles, government reports, policy briefs, and relevant grey literature that provide empirical or policy insights.	Non-peer-reviewed articles, and other publications that lack empirical data or policy analysis.

### Information sources

We searched MEDLINE, Embase, Global Health (all via Ovid), Web of Science Core Collection, SciELO (via Web of Science), and Global Index Medicus (all regional databases) on December 17, 2024 and included results from January 1, 2020 until November 30, 2024.

### Search strategy

The search strategy consisted of keywords and database-specific subject headings related to the following three concepts: climate change, dengue fever and LMICs. Please see supplemental file for full reproducible search strategies. Search terms related to climate change encompassed temperature, rainfall, humidity, and climate variability indicators commonly used in dengue research. Duplicate citations were manually removed with EndNote 20 (Clarivate Analytics, Philadelphia, PA) and then each database result was uploaded to Covidence (Veritas Health Innovation, Melbourne, Australia), a web-based systematic review platform, for screening and their system automatically identified and removed the remaining duplicate citations.

### Screening

All literature was imported into Covidence. The screening process was performed in two stages: 1) an initial review of titles and abstracts, followed by 2) examination of full texts. Seven authors from our research team conduct the screening. Screening was conducted to identify articles that meet our eligibility criteria as described in Table 1. Additionally, climate-related variables extracted included temperature, rainfall, humidity, and broader climate variability indicators. The senior author crosschecked results for accuracy for each stage.

### Data extraction

For all studies that met the eligibility criteria, data were extracted using a standardized form within Covidence and presented in Excel spreadsheets. Extracted variables mainly covered study design and methodology, participant characteristics, setting and context, interventions or exposures, and key findings. Six authors participated in the data extraction process, and all entries were subsequently cross-checked and revised by the senior author to ensure accuracy, consistency, and completeness of the information collected.

### Quality appraisal

The Mixed Methods Appraisal Tool (MMAT) [19] was used to assess the methodological quality of the included studies. The MMAT allows for the evaluation of a range of study designs, including qualitative, quantitative, and mixed methods studies. Each included study was evaluated for the suitability of its design in addressing our research question, the adequacy of the sample size, participants recruitment bias, outcome measurement accuracy, and the clarity of result reporting. A separate descriptive assessment was conducted for modeling studies that did not meet to the MMAT eligibility due to their simulation-based or computational design. A structured extraction approach was used to assess key information on model type, underlying assumptions, validation procedures, sensitivity or uncertainty analysis, and etc.

### Data synthesis

Extracted data were synthesized narratively using a thematic analysis approach. Studies were grouped by themes relevant to our research question, including climatic influences on dengue, urban and environmental factors, geographic and seasonal shifts in disease patterns, and government responses.

## Results

### Study selection

**Fig 1:**
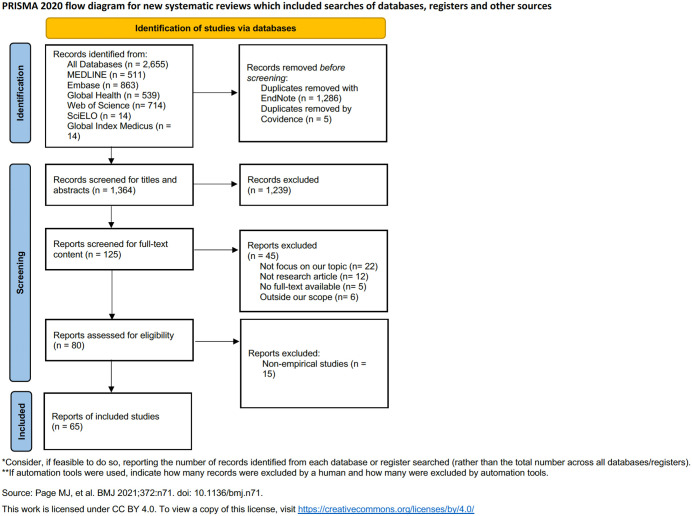
PRISMA Flow Diagram.

Our initial database search retrieved 1,359 records, of which five duplicates were removed before screening. The remaining 1,354 records were screened by title and abstract, resulting in the exclusion of 1,230 studies that did not meet the inclusion criteria. A total of 124 full-text articles were then reviewed, leading to the exclusion of 44 studies. Of the 80 articles assessed for eligibility, 15 were excluded for being non-empirical design. In total 65 studies met all inclusion criteria and were included in synthesis. Our detailed study selection process is summarized in Fig 1.

### Study characteristics

A total of 65 empirical studies were included in our final synthesis. Data were extracted using Covidence and organized into two tables. S1 Table summarizes general study characteristics, including geographic location, study aims, design type, and study period. Although dengue burden is substantial in parts of Latin America and sub-Saharan Africa, the majority of included studies were concentrated in South and Southeast Asia. This reflects regional differences in research output and data availability rather than the global distribution of dengue risk. S2 Table captures more technical details such as data sources, inclusion criteria, population descriptions, variables related to climate and urbanization, statistical methods used, and key findings. The extraction focused on variables relevant to our review objectives, particularly those focusing on dengue epidemiology in low- and middle-income countries.

**Fig 2:**
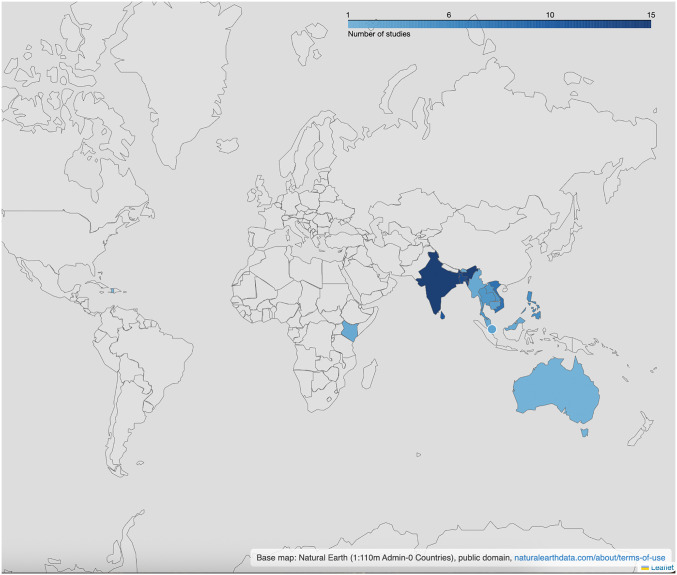
Geographical Distribution of Included Studies. Access to base layer of map: Natural Earth. Admin 0 – Countries, 1:110m cultural vectors. Public domain. Available from: https://www.naturalearthdata.com/downloads/110m-cultural-vectors/ (terms of use: https://www.naturalearthdata.com/about/terms-of-use/).

The included studies were published between 2020 and 2024, indicating the recent and growing interest in dengue-related research under changing climatic and urban conditions. This time window was intentionally selected to align with the COVID-19 era, during which significant disruptions occurred in healthcare systems, surveillance capacity, and urban mobility patterns. Rather than providing a comprehensive historical synthesis of climate–dengue relationships, this review aimed to examine recent evidence generated within this distinct global context.. All studies were conducted primarily in low- and middle-income countries, with India, Bangladesh, Sri Lanka, Vietnam, and the Philippines among the most frequently represented settings (Fig 2). The majority studies used retrospective ecological or time-series designs, predictive modeling, or spatial-temporal analyses. A smaller subset of studies used process-based models or hybrid methods that incorporated both climatic and demographic parameters. Most studies drew on routine surveillance data, meteorological datasets, and administrative health records.

Fig 2 illustrates the spatial distribution of studies included in the review, with a colour gradient representing the number of studies conducted in each country. The figure highlights a strong concentration of research in South and Southeast Asia, particularly India, Bangladesh, Sri Lanka, and Vietnam, while comparatively few studies were conducted outside these areas.

### Quality assessment

The methodological quality of the 50 included empirical studies was assessed using the MMAT tool, with the results presented in S3 Table. We evaluated studies employing quantitative descriptive designs (n = 18), quantitative non-randomized designs (n = 31), and one mixed methods design. Twelve studies, representing 24% of the sample, met all five MMAT criteria, indicating a high degree of methodological rigor across research design, data collection, and analysis. Twenty-four studies (48%) fulfilled four criteria, typically lacking detail in one aspect such as the treatment of missing data or the handling of confounding variables. Nine studies (18%) met three criteria, while five studies (10%) met two. Lower-scoring studies showed limited transparency in sampling procedures, insufficient justification for analytical methods, or incomplete reporting of methodological processes. No included studies were excluded based on MMAT results.

For the 15 included modeling studies that were not appraised using the MMAT tool, we assessed them descriptively using a structured framework (S4 Table). All 15 studies (100%) clearly reported their modeling objectives, and 13 (87%) provided sufficient detail on input data sources, which included climate observations and dengue surveillance data. Twelve studies (80%) explicitly described core model assumptions, such as vector-host interactions or climate-lag structures, while 11 (73%) reported conducting some form of sensitivity or uncertainty analysis. Validation procedures were described in 10 studies (67%), commonly using historical back-testing or cross-validation. Only four studies (27%) provided access to modeling code or computational tools. Despite varying levels of transparency, 14 studies (93%) reported implications for public health decision-making, including early warning systems, climate risk forecasting, and spatial risk mapping.

### Climatic influences on dengue

Temperature was the most consistently reported climatic condition, with 42 of the 65 studies (65%) identifying a positive relationship between rising ambient temperatures and increased dengue transmission [20–61]. Warmer conditions were associated with accelerated viral replication, higher mosquito biting rates, and extended transmission seasons [20,62–64]. Several modeling studies described a non-linear association, where transmission intensified within an optimal thermal range but declined beyond critical heat thresholds [62,65].

Rainfall was examined in 36 studies (55%) and generally associated with increased dengue risk through the creation of breeding habitats [20–23,25–29,32–42,45–53,56,59,63,66–69]. However, some studies noted mixed effects depending on rainfall intensity, infrastructure, and local water storage practices [20,22,23,25]. Humidity, while less frequently analyzed, was found to amplify mosquito activity, especially in combination with high temperatures [20,22,25,27,42,50,56,63,66]. Many studies also incorporated lag structures to reflect delayed effects of climatic exposures, ranging from one to twelve weeks [21,24,27,39,48,51,52,66]. Seasonal patterns consistently corresponded with monsoon or wet periods, particularly in tropical regions [47,49,62,63,69,70], highlighting the need for locally adapted climate-disease policies.

### Urbanization and built environment factors

Urbanization emerged as a significant factor for dengue transmission in LMICs, investigated in 23 of the 65 included studies (35%) [21,22,25,28,30–32,35,40,46,53,55,61,63,66–68,71–76]. Many studies identified unplanned urban growth, population density, and inadequate water or sanitation infrastructure as critical enablers of mosquito breeding and sustained transmission [21,22,63]. Informal settlements, in particular, were repeatedly cited for their role in creating conducive environments for vector proliferation due to limited access to piped water, waste management, and reliable drainage systems [66,71]. Several modeling and spatial studies highlighted the clustering of dengue incidence in peri-urban or transitioning zones, where rapid land-use change and insufficient public health infrastructure merged [21,41,46,54,77].

The effects of urban heat islands (UHIs) were examined, showing that surface temperature irregularity in densely populated cities could amplify vector activity and disease risk [25]. Additional studies found that high ambient temperatures in urban environments further increased transmission risk, even after adjusting for broader climatic trends [31,63]. These findings suggest that urban design such as green space availability and building density directly affect dengue transmission [46]. Changes in land use, such as deforestation and the expansion of urban development into previously vegetated areas, were associated with the emergence of new dengue transmission zones, particularly in regions of Latin America and Southeast Asia [51,54,78].

### Geographic expansion and seasonal shifts

Several studies reported the emergence of dengue in areas that were previously considered low risk or unaffected, including highland and temperate regions, as rising temperatures enabled mosquito vectors to survive in new environments [22,25,26,28,29,36,54,66]. Spatial modeling studies found that dengue risk is expanding into higher altitude and more rural regions, where public health infrastructure and vector control programs are often less developed [22,28,29,31,52,66,72]. These geographic shifts were obvious in countries experiencing rapid deforestation and the spread of urban development into previously vegetated areas [31,51,54,76]. In addition, multiple studies reported notable changes in dengue seasonality, including earlier onset, longer transmission periods, and the appearance of two seasonal peaks that corresponded with shifting rainfall and temperature patterns [20,28,38,40,41,54,60,62]. Studies from South and Southeast Asia frequently observed that the dengue transmission period extended beyond the traditional rainy season, with some areas now reporting outbreaks throughout most of the year [28,44,54,60,62].

### Government response

Government responses to the growing burden of climate-sensitive dengue were described in 19 of the 65 included studies (29%) [32–37,39–45,54,66,67,71,73,74]. Although this theme appeared in a smaller proportion of the literature compared to climatic and environmental drivers, the studies that did address government action provide important insight into how public health systems are responding to climate-related dengue risks. These studies documented a range of strategies, including vector control programs, community-based awareness campaigns, and early warning systems that integrated meteorological data with dengue surveillance [25,32,38,41,66,72,73]. Several countries had implemented predictive models or risk maps to guide local interventions, particularly during peak transmission seasons [48,50,64,71,78]. However, while these efforts reflected growing recognition of climate-related health risks, their implementation often faced operational and structural challenges. Common limitations included fragmented data systems, weak coordination between health and environmental agencies, and limited resources at civic level [22,32]. Many studies noted that early warning tools were still in pilot phases or lacked integration into routine health decision-making [34,36,41,43,69]. Several studies assessed whether these interventions were effective using measurable outcomes, indicating a gap in evidence about what actually works in real-world settings [26,29,36,37,40,59,64,74].

## Discussion

Our findings explore how climate variability and urban development are reshaping patterns of dengue transmission in LMICs. Higher temperatures, changing rainfall patterns, and the intensification of urban heat islands are contributing to longer transmission seasons and the spread of dengue into regions where it was previously uncommon [20,25,28,38,54,63]. Temperature was the most consistent climatic factor associated with dengue, but rainfall and humidity also played important roles, often interacting in ways that varied by setting [20,28,54]. The expansion of dengue into highland and temperate areas suggests that rising temperatures are creating new ecological conditions for disease transmission [25,28,36,52]. These shifts pose challenges for local health systems, many of which lack sufficient capacity to detect and respond to outbreaks in newly affected areas [40,54,69,74].

Unplanned urban growth also emerged as a key contributor to increased dengue risk, especially in communities with limited access to basic services such as water, sanitation, and waste management [22,28,63,74]. Studies identified informal settlements as consistent hotspots for dengue transmission, often associated with poor housing, inadequate waste disposal, and deteriorating environmental conditions [21,31,40,75]. Although some governments have introduced promising strategies such as early warning systems and risk mapping tools, these efforts are frequently limited in scope, poorly coordinated, or lacking sustainable funding support [32,41,67,74]. There remains limited documentation of full-scale implementation into routine public health planning. This lack of evidence indicates an urgent need for more applied research that examines not just what interventions are being introduced, but how effective they are in practice [26,29,31,36,40,59]. Although vaccination was not addressed in our included studies, emerging tools such as the TA-003 dengue vaccine [79] may become relevant to future response strategies in high-burden settings, particularly where policy supports its introduction.

This rapid review provides timely and policy insights into how climate change and urbanization are reshaping dengue transmission dynamics in LMICs. We highlight clear geographic and seasonal shifts in disease burden, identify vulnerable populations most at risk, and synthesize evidence on governmental responses to climate related health threats. By thematically organizing findings across climatic and urban risk factors, public agency responses, our review offers a valuable foundation for strengthening climate-informed surveillance systems, risk forecasting, and neglected tropical disease control strategies.

There are several limitations to acknowledge. As a rapid review, its scope was constrained by time-sensitive search and screening processes, which may have excluded relevant studies not captured in the search strategy. Additionally, the geographic distribution of included studies was not fully representative of the global burden of dengue. Although regions such as Latin America and parts of sub-Saharan Africa experience substantial dengue transmission, the majority of included studies in this review were concentrated in South and Southeast Asia. The observed imbalance likely reflects structural factors such as uneven research funding, data availability, and publication output across regions rather than true differences in disease importance. Moreover, the limited representation of governmental actions in the included studies not only restricted assessment of policy and programmatic responses but also highlights an important gap in the literature. Only English-language articles were included, which may have potentially introduced language bias and contributed to underrepresentation of relevant studies from Spanish- and Portuguese-speaking settings. The heterogeneity of study designs, geographic focus, and outcome measures limited our ability to conduct quantitative synthesis or meta-analysis.

## Conclusion

We underscore the importance of short-term weather variability and urbanization in shaping the spatial and temporal dynamics of dengue transmission in LMICs. Higher temperatures, rainfall variability, and expanding urban environments were commonly associated with changes in transmission patterns, transmission seasons, geographic risk, and control challenges in already resource-limited regions. While some governments have introduced innovative response strategies, systemic challenges in surveillance, coordination, and community engagement persist. Strengthening early warning systems, integrating climate and weather data into vector-born infectious diseases planning, and investing in sustainable urban infrastructure will be critical to mitigating the future burden of dengue fever, particularly as climate change may further influence these dynamics.

## Supporting information

S1 Table Data Extraction Study Characteristics. (XLSX)

S2 Table Data Extraction Study Outcomes. (XLSX)

S3 Table MMAT Appraisal Result. (DOCX)

S4 Table Descriptive Assessment. (XLSX)

S1 Text PRISMA 2020 Checklist. (DOCX)

S2 Text Search Strategy. (DOCX)
