# Supplementary material for: Battling dengue in a warming world: How climate and urbanization shape transmission in low- and middle-income countries (a rapid review)
Source: PLoS Negl Trop Dis. 2026 Jul 20;20(7):e0013758. doi: 10.1371/journal.pntd.0013758 (PMC13405114; doi:10.1371/journal.pntd.0013758)
Supplement: S3 Table — (DOCX) [file pntd.0013758.s003.docx]

| **First author (year)** | **Study design** | **MMAT appraisal** | **MMAT Scoring** |
| --- | --- | --- | --- |
| Bajwala (2020) | Quantitative Descriptive Study | Quantitative descriptive study: “4.1 Yes, 4.2 Yes, 4.3 Yes, 4.4 Can’t tell, 4.5 Yes” | ●●●●○ |
| Blanco (2022) | Quantitative Non-randomized Study | Quantitative Non-randomized Study: “3.1 Yes, 3.2 Yes, 3.3 Yes, 3.4 Yes, 3.5 Yes” | ●●●●● |
| Borah (2022) | Quantitative Non-randomized Study | Quantitative Non-randomized Study: “3.1 Yes, 3.2 Yes, 3.3 Yes, 3.4 Can’t tell, 3.5 Yes” | ●●●●○ |
| Brook (2024) | Quantitative Non-randomized Study | Quantitative Non-randomized Study: “3.1 Yes, 3.2 Yes, 3.3 Can’t tell, 3.4 No, 3.5 Yes” | ●●●○○ |
| Cheng (2020) | Quantitative Non-randomized Study | Quantitative Non-randomized Study: “3.1 Yes, 3.2 Yes, 3.3 Yes, 3.4 No, 3.5 Yes” | ●●●●○ |
| Colon-Gonzalez (2021) | Quantitative Non-randomized Study | Quantitative Non-randomized Study: “3.1 Yes, 3.2 Yes, 3.3 Yes, 3.4 No, 3.5 Yes” | ●●●●○ |
| Diouf (2024) | Quantitative Non-randomized Study | Quantitative Non-randomized Study: “3.1 Yes, 3.2 Yes, 3.3 Yes, 3.4 No, 3.5 Yes” | ●●●●○ |
| Dutta (2021) | Quantitative Non-randomized Study | Quantitative Non-randomized Study: “3.1 Yes, 3.2 Yes, 3.3 Yes, 3.4 No, 3.5 Yes” | ●●●●○ |
| Edillo (2022) | Quantitative Non-randomized Study | Quantitative Non-randomized Study: “3.1 Yes, 3.2 Yes, 3.3 Yes, 3.4 Yes, 3.5 Yes” | ●●●●● |
| Edillo (2024) | Quantitative Non-randomized Study | Quantitative Non-randomized Study: “3.1 Yes, 3.2 Yes, 3.3 Yes, 3.4 Yes, 3.5 Yes” | ●●●●● |
| Erandi (2021) | Quantitative Non-randomized Study | Quantitative Non-randomized Study: “3.1 Yes, 3.2 Yes, 3.3 Yes, 3.4 Can’t tell, 3.5 Yes” | ●●●●○ |
| Francisco (2021) | Quantitative Non-randomized Study | Quantitative Non-randomized Study: “3.1 Yes, 3.2 Yes, 3.3 No, 3.4 Can’t tell, 3.5 Yes” | ●●●○○ |
| Gibb (2023) | Quantitative Non-randomized Study | Quantitative Non-randomized Study: “3.1 Yes, 3.2 Yes, 3.3 Yes, 3.4 Yes, 3.5 Can’t tell” | ●●●●○ |
| Hasan (2024) | Quantitative Non-randomized Study | Quantitative Non-randomized Study: “3.1 Yes, 3.2 Yes, 3.3 No, 3.4 No, 3.5 Can’t tell” | ●●○○○ |
| Herath (2022) | Quantitative Non-randomized Study | Quantitative Non-randomized Study: “3.1 Yes, 3.2 Yes, 3.3 Yes, 3.4 No, 3.5 Can’t tell” | ●●●○○ |
| Hossain (2024) | Quantitative Non-randomized Study | Quantitative Non-randomized Study: “3.1 Yes, 3.2 Yes, 3.3 Can’t tell, 3.4 No, 3.5 Can’t tell” | ●●○○○ |
| Hossain (2023a) | Quantitative Non-randomized Study | Quantitative Non-randomized Study: “3.1 Yes, 3.2 Yes, 3.3 Can’t tell, 3.4 No, 3.5 Can’t tell” | ●●○○○ |
| Hossain (2023b) | Quantitative Non-randomized Study | Quantitative Non-randomized Study: “3.1 Yes, 3.2 Yes, 3.3 Yes, 3.4 No, 3.5 Can’t tell” | ●●●○○ |
| Kamau (2023) | Quantitative Descriptive Study | Quantitative descriptive study: “4.1 Yes, 4.2 Yes, 4.3 Yes, 4.4 Can’t tell, 4.5 Yes” | ●●●●○ |
| Miah (2024) | Quantitative Descriptive Study | Quantitative descriptive study: “4.1 Yes, 4.2 Yes, 4.3 Yes, 4.4 Can’t tell, 4.5 Yes” | ●●●●○ |
| Nguyen (2020) | Quantitative Descriptive Study | Quantitative descriptive study: “4.1 Yes, 4.2 Yes, 4.3 Yes, 4.4 Can’t tell, 4.5 Yes” | ●●●●○ |
| Nosrat (2020) | Quantitative Non-randomized Study | Quantitative Non-randomized Study: “3.1 Yes, 3.2 Yes, 3.3 Yes, 3.4 No, 3.5 Can’t tell” | ●●●○○ |
| Parker (2023) | Quantitative Non-randomized Study | Quantitative Non-randomized Study: “3.1 Yes, 3.2 Yes, 3.3 Can’t tell, 3.4 No, 3.5 Can’t tell” | ●●○○○ |
| Prabodanie (2020) | Quantitative Non-randomized Study | Quantitative Non-randomized Study: “3.1 Yes, 3.2 Yes, 3.3 Can’t tell, 3.4 No, 3.5 Yes” | ●●●○○ |
| Pramanik (2020) | Quantitative Descriptive Study | Quantitative descriptive study: “4.1 Yes, 4.2 Yes, 4.3 Yes, 4.4 Can’t tell, 4.5 Yes” | ●●●●○ |
| Rahman (2021) | Mixed Methods Study: Qualitative Study + Quantitative Descriptive Study | “Qualitative Study 1.1 Yes, 1.2 Yes, 1.3 Yes, 1.4 Yes, 1.5 Yes; Quantitative Descriptive Study 4.1 Yes, 4.2 Can’t tell, 4.3 Yes, 4.4 Can’t tell, 4.5 Yes; Mixed Methods Study 5.1 Yes, 5.2 Yes, 5.3 Yes, 5.4 Yes, 5.5 Yes.” | ●●●●● |
| Roy (2024) | Quantitative Descriptive Study | Quantitative descriptive study: “4.1 Yes, 4.2 Can’t tell, 4.3 Yes, 4.4 Can’t tell, 4.5 Yes” | ●●●○○ |
| Sanyal (2021) | Quantitative Descriptive Study | Quantitative descriptive study: “4.1 Yes, 4.2 Can’t tell, 4.3 Can’t tell, 4.4 Yes, 4.5 Can’t tell” | ●●○○○ |
| Sargent (2022) | Quantitative Descriptive Study | Quantitative descriptive study: “4.1 Yes, 4.2 Yes, 4.3 Yes, 4.4 Yes, 4.5 Yes” | ●●●●● |
| Sarma (2022) | Quantitative Non-randomized Study | Quantitative Non-randomized Study: “3.1 Yes, 3.2 Yes, 3.3 Can’t tell, 3.4 Yes, 3.5 Yes” | ●●●●○ |
| Shankar (2024) | Quantitative Descriptive Study | Quantitative descriptive study: “4.1 Yes, 4.2 Yes, 4.3 Yes, 4.4 Can’t tell, 4.5 Yes” | ●●●●○ |
| Shil (2020) | Quantitative Non-randomized Study | Quantitative Non-randomized Study: “3.1 Yes, 3.2 Yes, 3.3 Yes, 3.4 Yes, 3.5 Can’t tell” | ●●●●○ |
| Soukavong (2024) | Quantitative Non-randomized Study | Quantitative Non-randomized Study: “3.1 Yes, 3.2 Yes, 3.3 Yes, 3.4 Yes, 3.5 Can’t tell” | ●●●●○ |
| Subarna (2024) | Quantitative Descriptive Study | Quantitative descriptive study: “4.1 Yes, 4.2 Yes, 4.3 Yes, 4.4 Yes, 4.5 Yes” | ●●●●● |
| Sugeno (2023) | Quantitative Non-randomized Study | Quantitative Non-randomized Study: “3.1 Yes, 3.2 Yes, 3.3 Yes, 3.4 Yes, 3.5 Can’t tell” | ●●●●○ |
| Surendran (2022) | Quantitative Non-randomized Study | Quantitative Non-randomized Study: “3.1 Yes, 3.2 Yes, 3.3 Yes, 3.4 Yes, 3.5 Yes” | ●●●●● |
| Suresh (2023) | Quantitative Descriptive Study | Quantitative descriptive study: “4.1 Yes, 4.2 Yes, 4.3 Yes, 4.4 Yes, 4.5 Yes” | ●●●●● |
| Telle (2021) | Quantitative Non-randomized Study | Quantitative Non-randomized Study: “3.1 Yes, 3.2 Yes, 3.3 Yes, 3.4 Yes, 3.5 Can’t tell” | ●●●●○ |
| Tsheten (2020) | Quantitative Non-randomized Study | Quantitative Non-randomized Study: “3.1 Yes, 3.2 Yes, 3.3 Yes, 3.4 Yes, 3.5 Can’t tell” | ●●●●○ |
| Tsheten (2021) | Quantitative Non-randomized Study | Quantitative Non-randomized Study: “3.1 Yes, 3.2 Yes, 3.3 Yes, 3.4 Yes, 3.5 Can’t tell” | ●●●●○ |
| Tuan (2024) | Quantitative Non-randomized Study | Quantitative Non-randomized Study: “3.1 Yes, 3.2 Yes, 3.3 Yes, 3.4 Yes, 3.5 Can’t tell” | ●●●●○ |
| Udayanga (2020) | Quantitative Non-randomized Study | Quantitative Non-randomized Study: “3.1 Yes, 3.2 Yes, 3.3 Yes, 3.4 Yes, 3.5 Can’t tell” | ●●●●○ |
| Vaman (2024) | Quantitative Non-randomized Study | Quantitative Non-randomized Study: “3.1 Yes, 3.2 Yes, 3.3 Yes, 3.4 Yes, 3.5 Can’t tell” | ●●●●○ |
| Wagner (2020) | Quantitative Descriptive Study | Quantitative descriptive study: “4.1 Yes, 4.2 Yes, 4.3 Yes, 4.4 Yes, 4.5 Yes” | ●●●●● |
| Wang (2024a) | Quantitative Descriptive Study | Quantitative descriptive study: “4.1 Yes, 4.2 Can’t tell, 4.3 Yes, 4.4 Yes, 4.5 Yes” | ●●●●○ |
| Wang (2024b) | Quantitative Descriptive Study | Quantitative descriptive study: “4.1 Yes, 4.2 Can’t tell, 4.3 Yes, 4.4 Can’t tell, 4.5 Yes” | ●●●○○ |
| Xu (2020) | Quantitative Descriptive Study | Quantitative descriptive study: “4.1 Yes, 4.2 Yes, 4.3 Yes, 4.4 Yes, 4.5 Yes” | ●●●●● |
| Zafar (2021) | Quantitative Descriptive Study | Quantitative descriptive study: “4.1 Yes, 4.2 Yes, 4.3 Yes, 4.4 Yes, 4.5 Yes” | ●●●●● |
| Zaw (2023) | Quantitative Descriptive Study | Quantitative descriptive study: “4.1 Yes, 4.2 Can’t tell, 4.3 Yes, 4.4 Can’t tell, 4.5 Yes” | ●●●○○ |
| Zhang (2020) | Quantitative Descriptive Study | Quantitative descriptive study: “4.1 Yes, 4.2 Yes, 4.3 Yes, 4.4 Yes, 4.5 Yes” | ●●●●● |
