# Supplementary material for: Battling dengue in a warming world: How climate and urbanization shape transmission in low- and middle-income countries (a rapid review)
Source: PLoS Negl Trop Dis. 2026 Jul 20;20(7):e0013758. doi: 10.1371/journal.pntd.0013758 (PMC13405114; doi:10.1371/journal.pntd.0013758)
Supplement: S2 Text — (DOCX) [file pntd.0013758.s006.docx]

**Battling Dengue in a Warming World: Search Strategies**

**Databases and Strategies**

MEDLINE, Embase, Global Health, Web of Science, SciELO Index, and Global Index Medicus

| Ovid MEDLINE(R) ALL 1946 to December 16, 2024 | | |
| --- | --- | --- |
| **Date Searched** | 12/17/2024 | |
| **Concept** | **Search String** | **Results** |
| **1** | ((climate ADJ2 (chang* OR warm* OR variab*)) OR global warming OR ((warm* OR rising OR extreme) ADJ2 temperature*) OR extreme weather OR extreme heat OR rain* OR flood* OR urbanization OR urbanisation OR (urban ADJ2 (sprawl* OR expan*)) OR (population ADJ2 grow*)).ti,ab,kf. OR Climate Change/ OR Global Warming/ OR Extreme Weather/ OR Extreme Heat/ OR Temperature/ OR Rain/ OR Urbanization/ | 497,448 |
| **2** | (dengue OR break bone fever OR breakbone fever OR Singapore hemorrhagic fever OR Thai hemorrhagic fever OR Philippine hemorrhagic fever).ti,ab,kf. OR Dengue/ OR Severe Dengue/ | 31,570 |
| **3. LMICs** | (afghanistan OR albania OR algeria OR angola OR argentina OR armenia OR armenian OR azerbaijan OR bangladesh OR republic of belarus OR belarus OR byelarus OR belorussia OR byelorussian OR belize OR british honduras OR benin OR dahomey OR bhutan OR bolivia OR "bosnia and herzegovina" OR bosnia OR herzegovina OR botswana OR bechuanaland OR brazil OR brasil OR burkina faso OR burkina fasso OR upper volta OR burundi OR urundi OR cabo verde OR cape verde OR cambodia OR kampuchea OR khmer republic OR cameroon OR cameron OR cameroun OR central african republic OR ubangi shari OR chad OR china OR colombia OR comoros OR comoro islands OR iles comores OR mayotte OR democratic republic of the congo OR democratic republic congo OR congo OR zaire OR costa rica OR "cote d’ivoire" OR "cote d’ ivoire" OR cote divoire OR cote d ivoire OR ivory coast OR cuba OR djibouti OR french somaliland OR dominica OR dominican republic OR ecuador OR egypt OR united arab republic OR el salvador OR equatorial guinea OR spanish guinea OR eritrea OR eswatini OR swaziland OR ethiopia OR fiji OR gabon OR gabonese republic OR gambia OR "georgia (republic)" OR georgian OR ghana OR gold coast OR grenada OR guatemala OR guinea OR guinea bissau OR haiti OR hispaniola OR honduras OR india OR indonesia OR timor OR iran OR iraq OR jamaica OR jordan OR kazakhstan OR kazakh OR kenya OR "democratic people’s republic of korea" OR north korea OR korea OR kosovo OR kyrgyzstan OR kirghizia OR kirgizstan OR kyrgyz republic OR kirghiz OR laos OR lao pdr OR "lao people's democratic republic" OR lebanon OR lebanese republic OR lesotho OR basutoland OR liberia OR libya OR libyan arab jamahiriya OR republic of north macedonia OR macedonia OR madagascar OR malagasy republic OR malawi OR nyasaland OR malaysia OR malay federation OR malaya federation OR maldives OR indian ocean islands OR mali OR micronesia OR federated states of micronesia OR kiribati OR marshall islands OR nauru OR northern mariana islands OR palau OR tuvalu OR mauritania OR mauritius OR mexico OR moldova OR moldovian OR mongolia OR montenegro OR morocco OR ifni OR mozambique OR portuguese east africa OR myanmar OR burma OR namibia OR nepal OR netherlands antilles OR nicaragua OR niger OR nigeria OR muscat OR pakistan OR papua new guinea OR new guinea OR paraguay OR peru OR philippines OR philipines OR phillipines OR phillippines OR rwanda OR ruanda OR samoa OR pacific islands OR polynesia OR samoan islands OR navigator island OR navigator islands OR "sao tome and principe" OR senegal OR serbia OR sierra leone OR melanesia OR solomon island OR solomon islands OR norfolk island OR norfolk islands OR somalia OR south africa OR south sudan OR sri lanka OR ceylon OR saint lucia OR "st. lucia" OR "saint vincent and the grenadines" OR saint vincent OR "st. vincent" OR grenadines OR sudan OR suriname OR surinam OR dutch guiana OR netherlands guiana OR syria OR syrian arab republic OR tajikistan OR tadjikistan OR tadzhikistan OR tadzhik OR tanzania OR tanganyika OR thailand OR siam OR timor leste OR east timor OR togo OR togolese republic OR tonga OR tunisia OR turkey OR turkmenistan OR turkmen OR uganda OR ukraine OR uzbekistan OR uzbek OR vanuatu OR new hebrides OR vietnam OR viet nam OR middle east OR west bank OR gaza OR palestine OR yemen OR zambia OR zimbabwe OR northern rhodesia OR global south OR africa south of the sahara OR sub-saharan africa OR subsaharan africa OR central africa OR north africa OR northern africa OR magreb OR maghrib OR sahara OR southern africa OR east africa OR eastern africa OR west africa OR western africa OR west indies OR indian ocean islands OR caribbean OR central america OR latin america OR "south and central america" OR south america OR central asia OR north asia OR northern asia OR southeastern asia OR south eastern asia OR southeast asia OR south east asia OR western asia OR east europe OR eastern europe OR developing country OR developing countries OR developing nation? OR developing population? OR developing world OR less developed countr* OR less developed nation? OR less developed population? OR less developed world OR lesser developed countr* OR lesser developed nation? OR lesser developed population? OR lesser developed world OR under developed countr* OR under developed nation? OR under developed population? OR under developed world OR underdeveloped countr* OR underdeveloped nation? OR underdeveloped population? OR underdeveloped world OR middle income countr* OR middle income nation? OR middle income population? OR low income countr* OR low income nation? OR low income population? OR lower income countr* OR lower income nation? OR lower income population? OR underserved countr* OR underserved nation? OR underserved population? OR underserved world OR under served countr* OR under served nation? OR under served population? OR under served world OR deprived countr* OR deprived nation? OR deprived population? OR deprived world OR poor countr* OR poor nation? OR poor population? OR poor world OR poorer countr* OR poorer nation? OR poorer population? OR poorer world OR developing econom* OR less developed econom* OR lesser developed econom* OR under developed econom* OR underdeveloped econom* OR middle income econom* OR low income econom* OR lower income econom* OR low gdp OR low gnp OR low gross domestic OR low gross national OR lower gdp OR lower gnp OR lower gross domestic OR lower gross national OR lmic OR lmics OR third world OR lami countr* OR transitional countr* OR emerging economies OR emerging nation?).ti,ab,sh,kf. | 2,208,793 |
| **Combo** | 1 AND 2 AND 3 | 1359 |
| **Limits** | AND English.lg.  AND Limit [n] to dt=20200101-20251231 |  |
| **Total** |  | 511 |

| Ovid Embase Classic+Embase 1947 to 2024 December 16 | | |
| --- | --- | --- |
| **Date Searched** | 12/17/2024 | |
| **Concept** | **Search String** | **Results** |
| **1** | ((climate ADJ2  (chang* OR warm* OR variab*)) OR  global warming OR  ((warm* OR rising OR extreme) ADJ2 temperature*) OR  extreme weather OR  extreme heat OR  rain* OR  flood* OR  urbanization OR  urbanisation OR  (urban ADJ2 (sprawl* OR expan*)) OR  (population ADJ2 grow*)).ti,ab,kf. OR  Climate Change/ OR  Global Warming/ OR  Extreme Weather/ OR  Extreme Heat/ OR  Temperature/ OR  Rain/ OR  Urbanization/ | 615,439 |
| **2** | (dengue OR  break bone fever OR  breakbone fever OR  Singapore hemorrhagic fever OR  Thai hemorrhagic fever OR  Philippine hemorrhagic fever).ti,ab,kf. OR  Dengue/ OR  Severe Dengue/ | 44,809 |
| **3. LMICs** | (afghanistan OR albania OR algeria OR angola OR argentina OR armenia OR armenian OR azerbaijan OR bangladesh OR republic of belarus OR belarus OR byelarus OR belorussia OR byelorussian OR belize OR british honduras OR benin OR dahomey OR bhutan OR bolivia OR "bosnia and herzegovina" OR bosnia OR herzegovina OR botswana OR bechuanaland OR brazil OR brasil OR burkina faso OR burkina fasso OR upper volta OR burundi OR urundi OR cabo verde OR cape verde OR cambodia OR kampuchea OR khmer republic OR cameroon OR cameron OR cameroun OR central african republic OR ubangi shari OR chad OR china OR colombia OR comoros OR comoro islands OR iles comores OR mayotte OR democratic republic of the congo OR democratic republic congo OR congo OR zaire OR costa rica OR "cote d’ivoire" OR "cote d’ ivoire" OR cote divoire OR cote d ivoire OR ivory coast OR cuba OR djibouti OR french somaliland OR dominica OR dominican republic OR ecuador OR egypt OR united arab republic OR el salvador OR equatorial guinea OR spanish guinea OR eritrea OR eswatini OR swaziland OR ethiopia OR fiji OR gabon OR gabonese republic OR gambia OR "georgia (republic)" OR georgian OR ghana OR gold coast OR grenada OR guatemala OR guinea OR guinea bissau OR haiti OR hispaniola OR honduras OR india OR indonesia OR timor OR iran OR iraq OR jamaica OR jordan OR kazakhstan OR kazakh OR kenya OR "democratic people’s republic of korea" OR north korea OR korea OR kosovo OR kyrgyzstan OR kirghizia OR kirgizstan OR kyrgyz republic OR kirghiz OR laos OR lao pdr OR "lao people's democratic republic" OR lebanon OR lebanese republic OR lesotho OR basutoland OR liberia OR libya OR libyan arab jamahiriya OR republic of north macedonia OR macedonia OR madagascar OR malagasy republic OR malawi OR nyasaland OR malaysia OR malay federation OR malaya federation OR maldives OR indian ocean islands OR mali OR micronesia OR federated states of micronesia OR kiribati OR marshall islands OR nauru OR northern mariana islands OR palau OR tuvalu OR mauritania OR mauritius OR mexico OR moldova OR moldovian OR mongolia OR montenegro OR morocco OR ifni OR mozambique OR portuguese east africa OR myanmar OR burma OR namibia OR nepal OR netherlands antilles OR nicaragua OR niger OR nigeria OR muscat OR pakistan OR papua new guinea OR new guinea OR paraguay OR peru OR philippines OR philipines OR phillipines OR phillippines OR rwanda OR ruanda OR samoa OR pacific islands OR polynesia OR samoan islands OR navigator island OR navigator islands OR "sao tome and principe" OR senegal OR serbia OR sierra leone OR melanesia OR solomon island OR solomon islands OR norfolk island OR norfolk islands OR somalia OR south africa OR south sudan OR sri lanka OR ceylon OR saint lucia OR "st. lucia" OR "saint vincent and the grenadines" OR saint vincent OR "st. vincent" OR grenadines OR sudan OR suriname OR surinam OR dutch guiana OR netherlands guiana OR syria OR syrian arab republic OR tajikistan OR tadjikistan OR tadzhikistan OR tadzhik OR tanzania OR tanganyika OR thailand OR siam OR timor leste OR east timor OR togo OR togolese republic OR tonga OR tunisia OR turkey OR turkmenistan OR turkmen OR uganda OR ukraine OR uzbekistan OR uzbek OR vanuatu OR new hebrides OR vietnam OR viet nam OR middle east OR west bank OR gaza OR palestine OR yemen OR zambia OR zimbabwe OR northern rhodesia OR global south OR africa south of the sahara OR sub-saharan africa OR subsaharan africa OR central africa OR north africa OR northern africa OR magreb OR maghrib OR sahara OR southern africa OR east africa OR eastern africa OR west africa OR western africa OR west indies OR indian ocean islands OR caribbean OR central america OR latin america OR "south and central america" OR south america OR central asia OR north asia OR northern asia OR southeastern asia OR south eastern asia OR southeast asia OR south east asia OR western asia OR east europe OR eastern europe OR developing country OR developing countries OR developing nation? OR developing population? OR developing world OR less developed countr* OR less developed nation? OR less developed population? OR less developed world OR lesser developed countr* OR lesser developed nation? OR lesser developed population? OR lesser developed world OR under developed countr* OR under developed nation? OR under developed population? OR under developed world OR underdeveloped countr* OR underdeveloped nation? OR underdeveloped population? OR underdeveloped world OR middle income countr* OR middle income nation? OR middle income population? OR low income countr* OR low income nation? OR low income population? OR lower income countr* OR lower income nation? OR lower income population? OR underserved countr* OR underserved nation? OR underserved population? OR underserved world OR under served countr* OR under served nation? OR under served population? OR under served world OR deprived countr* OR deprived nation? OR deprived population? OR deprived world OR poor countr* OR poor nation? OR poor population? OR poor world OR poorer countr* OR poorer nation? OR poorer population? OR poorer world OR developing econom* OR less developed econom* OR lesser developed econom* OR under developed econom* OR underdeveloped econom* OR middle income econom* OR low income econom* OR lower income econom* OR low gdp OR low gnp OR low gross domestic OR low gross national OR lower gdp OR lower gnp OR lower gross domestic OR lower gross national OR lmic OR lmics OR third world OR lami countr* OR transitional countr* OR emerging economies OR emerging nation?).ti,ab,sh,kf. | 2,674,170 |
| **Combo** | 1 AND 2 AND 3 | 2,103 |
| **Limits** | AND English.lg.  AND Limit [n] to dc=20200101-20251231 |  |
| **Total** |  | 863 |

| Ovid Global Health 1973 to 2024 Week 50 | | |
| --- | --- | --- |
| **Date Searched** | 12/17/2024 | |
| **Concept** | **Search String** | **Results** |
| **1** | ((climate ADJ2  (chang* OR warm* OR variab*)) OR  global warming OR  ((warm* OR rising OR extreme) ADJ2 temperature*) OR  extreme weather OR  extreme heat OR  rain* OR  flood* OR  urbanization OR  urbanisation OR  (urban ADJ2 (sprawl* OR expan*)) OR  (population ADJ2 grow*)).ti,ab. OR  Climate Change/ OR  Global Warming/ OR  Greenhouse Effect/ OR  Rain/ OR  Urbanization/ | 70,506 |
| **2** | (dengue OR  break bone fever OR  breakbone fever OR  Singapore hemorrhagic fever OR  Thai hemorrhagic fever OR  Philippine hemorrhagic fever).ti,ab. OR  Dengue/ OR  Dengue Haemorrhagic Fever/ OR  Dengue Shock Syndrome/ | 28,167 |
| **3. LMICs** | (afghanistan OR albania OR algeria OR angola OR argentina OR armenia OR armenian OR azerbaijan OR bangladesh OR republic of belarus OR belarus OR byelarus OR belorussia OR byelorussian OR belize OR british honduras OR benin OR dahomey OR bhutan OR bolivia OR "bosnia and herzegovina" OR bosnia OR herzegovina OR botswana OR bechuanaland OR brazil OR brasil OR burkina faso OR burkina fasso OR upper volta OR burundi OR urundi OR cabo verde OR cape verde OR cambodia OR kampuchea OR khmer republic OR cameroon OR cameron OR cameroun OR central african republic OR ubangi shari OR chad OR china OR colombia OR comoros OR comoro islands OR iles comores OR mayotte OR democratic republic of the congo OR democratic republic congo OR congo OR zaire OR costa rica OR "cote d’ivoire" OR "cote d’ ivoire" OR cote divoire OR cote d ivoire OR ivory coast OR cuba OR djibouti OR french somaliland OR dominica OR dominican republic OR ecuador OR egypt OR united arab republic OR el salvador OR equatorial guinea OR spanish guinea OR eritrea OR eswatini OR swaziland OR ethiopia OR fiji OR gabon OR gabonese republic OR gambia OR "georgia (republic)" OR georgian OR ghana OR gold coast OR grenada OR guatemala OR guinea OR guinea bissau OR haiti OR hispaniola OR honduras OR india OR indonesia OR timor OR iran OR iraq OR jamaica OR jordan OR kazakhstan OR kazakh OR kenya OR "democratic people’s republic of korea" OR north korea OR korea OR kosovo OR kyrgyzstan OR kirghizia OR kirgizstan OR kyrgyz republic OR kirghiz OR laos OR lao pdr OR "lao people's democratic republic" OR lebanon OR lebanese republic OR lesotho OR basutoland OR liberia OR libya OR libyan arab jamahiriya OR republic of north macedonia OR macedonia OR madagascar OR malagasy republic OR malawi OR nyasaland OR malaysia OR malay federation OR malaya federation OR maldives OR indian ocean islands OR mali OR micronesia OR federated states of micronesia OR kiribati OR marshall islands OR nauru OR northern mariana islands OR palau OR tuvalu OR mauritania OR mauritius OR mexico OR moldova OR moldovian OR mongolia OR montenegro OR morocco OR ifni OR mozambique OR portuguese east africa OR myanmar OR burma OR namibia OR nepal OR netherlands antilles OR nicaragua OR niger OR nigeria OR muscat OR pakistan OR papua new guinea OR new guinea OR paraguay OR peru OR philippines OR philipines OR phillipines OR phillippines OR rwanda OR ruanda OR samoa OR pacific islands OR polynesia OR samoan islands OR navigator island OR navigator islands OR "sao tome and principe" OR senegal OR serbia OR sierra leone OR melanesia OR solomon island OR solomon islands OR norfolk island OR norfolk islands OR somalia OR south africa OR south sudan OR sri lanka OR ceylon OR saint lucia OR "st. lucia" OR "saint vincent and the grenadines" OR saint vincent OR "st. vincent" OR grenadines OR sudan OR suriname OR surinam OR dutch guiana OR netherlands guiana OR syria OR syrian arab republic OR tajikistan OR tadjikistan OR tadzhikistan OR tadzhik OR tanzania OR tanganyika OR thailand OR siam OR timor leste OR east timor OR togo OR togolese republic OR tonga OR tunisia OR turkey OR turkmenistan OR turkmen OR uganda OR ukraine OR uzbekistan OR uzbek OR vanuatu OR new hebrides OR vietnam OR viet nam OR middle east OR west bank OR gaza OR palestine OR yemen OR zambia OR zimbabwe OR northern rhodesia OR global south OR africa south of the sahara OR sub-saharan africa OR subsaharan africa OR central africa OR north africa OR northern africa OR magreb OR maghrib OR sahara OR southern africa OR east africa OR eastern africa OR west africa OR western africa OR west indies OR indian ocean islands OR caribbean OR central america OR latin america OR "south and central america" OR south america OR central asia OR north asia OR northern asia OR southeastern asia OR south eastern asia OR southeast asia OR south east asia OR western asia OR east europe OR eastern europe OR developing country OR developing countries OR developing nation? OR developing population? OR developing world OR less developed countr* OR less developed nation? OR less developed population? OR less developed world OR lesser developed countr* OR lesser developed nation? OR lesser developed population? OR lesser developed world OR under developed countr* OR under developed nation? OR under developed population? OR under developed world OR underdeveloped countr* OR underdeveloped nation? OR underdeveloped population? OR underdeveloped world OR middle income countr* OR middle income nation? OR middle income population? OR low income countr* OR low income nation? OR low income population? OR lower income countr* OR lower income nation? OR lower income population? OR underserved countr* OR underserved nation? OR underserved population? OR underserved world OR under served countr* OR under served nation? OR under served population? OR under served world OR deprived countr* OR deprived nation? OR deprived population? OR deprived world OR poor countr* OR poor nation? OR poor population? OR poor world OR poorer countr* OR poorer nation? OR poorer population? OR poorer world OR developing econom* OR less developed econom* OR lesser developed econom* OR under developed econom* OR underdeveloped econom* OR middle income econom* OR low income econom* OR lower income econom* OR low gdp OR low gnp OR low gross domestic OR low gross national OR lower gdp OR lower gnp OR lower gross domestic OR lower gross national OR lmic OR lmics OR third world OR lami countr* OR transitional countr* OR emerging economies OR emerging nation?).ti,ab,sh. | 1,400,140 |
| **Combo** | 1 AND 2 AND 3 | 1,660 |
| **Limits** | AND English.lg.  AND Limit [n] to yr=2020-2025 |  |
| **Total** |  | 539 |

| Web of Science Core Collection | | |
| --- | --- | --- |
| **Date Searched** | [12/17/2024](https://ovidsp.ovid.com/ovidweb.cgi?T=JS&NEWS=N&PAGE=main&SHAREDSEARCHID=55923ISVBVQxnscQSQPfuGfcRUSR2qQ5dddoNPYivAu7t3SPxxnaHOBp03niuai1z) | |
| **Concept** | **Search String** | **Results** |
| **1** | TS=((("climate" NEAR/2  ("chang*" OR "warm*" OR "variab*")) OR  "global warming" OR  (("warm*" OR "rising" OR "extreme") NEAR/2 "temperature*") OR  "extreme weather" OR  "extreme heat" OR  "rain*" OR  "flood*" OR  "urbanization" OR  "urbanisation" OR  ("urban" NEAR/2 ("sprawl*" OR "expan*")) OR  ("population" NEAR/2 "grow*"))) | 1,232,524 |
| **2** | TS=("dengue" OR  "break bone fever" OR  "breakbone fever" OR  "Singapore hemorrhagic fever" OR  "Thai hemorrhagic fever" OR  "Philippine hemorrhagic fever") | 39,596 |
| **3. LMICs** | TS=("afghanistan" OR  "albania" OR  "algeria" OR  "angola" OR  "argentina" OR  "armenia" OR  "armenian" OR  "azerbaijan" OR  "bangladesh" OR  "republic of belarus" OR  "belarus" OR  "byelarus" OR  "belorussia" OR  "byelorussian" OR  "belize" OR  "british honduras" OR  "benin" OR  "dahomey" OR  "bhutan" OR  "bolivia" OR  "bosnia and herzegovina" OR  "bosnia" OR  "herzegovina" OR  "botswana" OR  "bechuanaland" OR  "brazil" OR  "brasil" OR  "burkina faso" OR  "burkina fasso" OR  "upper volta" OR  "burundi" OR  "urundi" OR  "cabo verde" OR  "cape verde" OR  "cambodia" OR  "kampuchea" OR  "khmer republic" OR  "cameroon" OR  "cameron" OR  "cameroun" OR  "central african republic" OR  "ubangi shari" OR  "chad" OR  "china" OR  "colombia" OR  "comoros" OR  "comoro islands" OR  "iles comores" OR  "mayotte" OR  "democratic republic of the congo" OR  "democratic republic congo" OR  "congo" OR  "zaire" OR  "costa rica" OR  "cote d’ivoire" OR  "cote d’ ivoire" OR  "cote divoire" OR  "cote d ivoire" OR  "ivory coast" OR  "cuba" OR  "djibouti" OR  "french somaliland" OR  "dominica" OR  "dominican republic" OR  "ecuador" OR  "egypt" OR  "united arab republic" OR  "el salvador" OR  "equatorial guinea" OR  "spanish guinea" OR  "eritrea" OR  "eswatini" OR  "swaziland" OR  "ethiopia" OR  "fiji" OR  "gabon" OR  "gabonese republic" OR  "gambia" OR  "georgia (republic)" OR  "georgian" OR  "ghana" OR  "gold coast" OR  "grenada" OR  "guatemala" OR  "guinea" OR  "guinea bissau" OR  "haiti" OR  "hispaniola" OR  "honduras" OR  "india" OR  "indonesia" OR  "timor" OR  "iran" OR  "iraq" OR  "jamaica" OR  "jordan" OR  "kazakhstan" OR  "kazakh" OR  "kenya" OR  "democratic people’s republic of korea" OR  "north korea" OR  "korea" OR  "kosovo" OR  "kyrgyzstan" OR  "kirghizia" OR  "kirgizstan" OR  "kyrgyz republic" OR  "kirghiz" OR  "laos" OR  "lao pdr" OR  "lao people's democratic republic" OR  "lebanon" OR  "lebanese republic" OR  "lesotho" OR  "basutoland" OR  "liberia" OR  "libya" OR  "libyan arab jamahiriya" OR  "republic of north macedonia" OR  "macedonia" OR  "madagascar" OR  "malagasy republic" OR  "malawi" OR  "nyasaland" OR  "malaysia" OR  "malay federation" OR  "malaya federation" OR  "maldives" OR  "indian ocean islands" OR  "mali" OR  "micronesia" OR  "federated states of micronesia" OR  "kiribati" OR  "marshall islands" OR  "nauru" OR  "northern mariana islands" OR  "palau" OR  "tuvalu" OR  "mauritania" OR  "mauritius" OR  "mexico" OR  "moldova" OR  "moldovian" OR  "mongolia" OR  "montenegro" OR  "morocco" OR  "ifni" OR  "mozambique" OR  "portuguese east africa" OR  "myanmar" OR  "burma" OR  "namibia" OR  "nepal" OR  "netherlands antilles" OR  "nicaragua" OR  "niger" OR  "nigeria" OR  "muscat" OR  "pakistan" OR  "papua new guinea" OR  "new guinea" OR  "paraguay" OR  "peru" OR  "philippines" OR  "philipines" OR  "phillipines" OR  "phillippines" OR  "rwanda" OR  "ruanda" OR  "samoa" OR  "pacific islands" OR  "polynesia" OR  "samoan islands" OR  "navigator island" OR  "navigator islands" OR  "sao tome and principe" OR  "senegal" OR  "serbia" OR  "sierra leone" OR  "melanesia" OR  "solomon island" OR  "solomon islands" OR  "norfolk island" OR  "norfolk islands" OR  "somalia" OR  "south africa" OR  "south sudan" OR  "sri lanka" OR  "ceylon" OR  "saint lucia" OR  "st. lucia" OR  "saint vincent and the grenadines" OR  "saint vincent" OR  "st. vincent" OR  "grenadines" OR  "sudan" OR  "suriname" OR  "surinam" OR  "dutch guiana" OR  "netherlands guiana" OR  "syria" OR  "syrian arab republic" OR  "tajikistan" OR  "tadjikistan" OR  "tadzhikistan" OR  "tadzhik" OR  "tanzania" OR  "tanganyika" OR  "thailand" OR  "siam" OR  "timor leste" OR  "east timor" OR  "togo" OR  "togolese republic" OR  "tonga" OR  "tunisia" OR  "turkey" OR  "turkmenistan" OR  "turkmen" OR  "uganda" OR  "ukraine" OR  "uzbekistan" OR  "uzbek" OR  "vanuatu" OR  "new hebrides" OR  "vietnam" OR  "viet nam" OR  "middle east" OR  "west bank" OR  "gaza" OR  "palestine" OR  "yemen" OR  "zambia" OR  "zimbabwe" OR  "northern rhodesia" OR  "global south" OR  "africa south of the sahara" OR  "sub-saharan africa" OR  "subsaharan africa" OR  "central africa" OR  "north africa" OR  "northern africa" OR  "magreb" OR  "maghrib" OR  "sahara" OR  "southern africa" OR  "east africa" OR  "eastern africa" OR  "west africa" OR  "western africa" OR  "west indies" OR  "caribbean" OR  "central america" OR  "latin america" OR  "south and central america" OR  "south america" OR  "central asia" OR  "north asia" OR  "northern asia" OR  "southeastern asia" OR  "south eastern asia" OR  "southeast asia" OR  "south east asia" OR  "western asia" OR  "east europe" OR  "eastern europe" OR  "developing country" OR  "developing countries" OR  "developing nation?" OR  "developing population?" OR  "developing world" OR  "less developed countr*" OR  "less developed nation?" OR  "less developed population?" OR  "less developed world" OR  "lesser developed countr*" OR  "lesser developed nation?" OR  "lesser developed population?" OR  "lesser developed world" OR  "under developed countr*" OR  "under developed nation?" OR  "under developed population?" OR  "under developed world" OR  "underdeveloped countr*" OR  "underdeveloped nation?" OR  "underdeveloped population?" OR  "underdeveloped world" OR  "middle income countr*" OR  "middle income nation?" OR  "middle income population?" OR  "low income countr*" OR  "low income nation?" OR  "low income population?" OR  "lower income countr*" OR  "lower income nation?" OR  "lower income population?" OR  "underserved countr*" OR  "underserved nation?" OR  "underserved population?" OR  "underserved world" OR  "under served countr*" OR  "under served nation?" OR  "under served population?" OR  "under served world" OR  "deprived countr*" OR  "deprived nation?" OR  "deprived population?" OR  "deprived world" OR  "poor countr*" OR  "poor nation?" OR  "poor population?" OR  "poor world" OR  "poorer countr*" OR  "poorer nation?" OR  "poorer population?" OR  "poorer world" OR  "developing econom*" OR  "less developed econom*" OR  "lesser developed econom*" OR  "under developed econom*" OR  "underdeveloped econom*" OR  "middle income econom*" OR  "low income econom*" OR  "lower income econom*" OR  "low gdp" OR  "low gnp" OR  "low gross domestic" OR  "low gross national" OR  "lower gdp" OR  "lower gnp" OR  "lower gross domestic" OR  "lower gross national" OR  "lmic" OR  "lmics" OR  "third world" OR  "lami countr*" OR  "transitional countr*" OR  "emerging economies" OR  "emerging nation?") | 4,562,707 |
| **Combo** | 1 AND 2 AND 3 | 1,725 |
| **Limits** | AND English Language  AND DOP=2020-01-01/2025-12-31 |  |
| **Total** |  | 714 |

**<<>><<>><<>><<>><<>> Search Updates <<>><<>><<>><<>><<>>**

| SciELO Citation Index (via Web of Science) | | |
| --- | --- | --- |
| **Date Searched** | 2/23/2026 | |
| **Concept** | **Search String** | **Results** |
| **1** | TS=((("climate" NEAR/2  ("chang*" OR "warm*" OR "variab*")) OR  "global warming" OR  (("warm*" OR "rising" OR "extreme") NEAR/2 "temperature*") OR  "extreme weather" OR  "extreme heat" OR  "rain*" OR  "flood*" OR  "urbanization" OR  "urbanisation" OR  ("urban" NEAR/2 ("sprawl*" OR "expan*")) OR  ("population" NEAR/2 "grow*"))) | 26,965 |
| **2** | TS=("dengue" OR  "break bone fever" OR  "breakbone fever" OR  "Singapore hemorrhagic fever" OR  "Thai hemorrhagic fever" OR  "Philippine hemorrhagic fever") | 2,380 |
| **3. LMICs** | TS=("afghanistan" OR  "albania" OR  "algeria" OR  "angola" OR  "argentina" OR  "armenia" OR  "armenian" OR  "azerbaijan" OR  "bangladesh" OR  "republic of belarus" OR  "belarus" OR  "byelarus" OR  "belorussia" OR  "byelorussian" OR  "belize" OR  "british honduras" OR  "benin" OR  "dahomey" OR  "bhutan" OR  "bolivia" OR  "bosnia and herzegovina" OR  "bosnia" OR  "herzegovina" OR  "botswana" OR  "bechuanaland" OR  "brazil" OR  "brasil" OR  "burkina faso" OR  "burkina fasso" OR  "upper volta" OR  "burundi" OR  "urundi" OR  "cabo verde" OR  "cape verde" OR  "cambodia" OR  "kampuchea" OR  "khmer republic" OR  "cameroon" OR  "cameron" OR  "cameroun" OR  "central african republic" OR  "ubangi shari" OR  "chad" OR  "china" OR  "colombia" OR  "comoros" OR  "comoro islands" OR  "iles comores" OR  "mayotte" OR  "democratic republic of the congo" OR  "democratic republic congo" OR  "congo" OR  "zaire" OR  "costa rica" OR  "cote d’ivoire" OR  "cote d’ ivoire" OR  "cote divoire" OR  "cote d ivoire" OR  "ivory coast" OR  "cuba" OR  "djibouti" OR  "french somaliland" OR  "dominica" OR  "dominican republic" OR  "ecuador" OR  "egypt" OR  "united arab republic" OR  "el salvador" OR  "equatorial guinea" OR  "spanish guinea" OR  "eritrea" OR  "eswatini" OR  "swaziland" OR  "ethiopia" OR  "fiji" OR  "gabon" OR  "gabonese republic" OR  "gambia" OR  "georgia (republic)" OR  "georgian" OR  "ghana" OR  "gold coast" OR  "grenada" OR  "guatemala" OR  "guinea" OR  "guinea bissau" OR  "haiti" OR  "hispaniola" OR  "honduras" OR  "india" OR  "indonesia" OR  "timor" OR  "iran" OR  "iraq" OR  "jamaica" OR  "jordan" OR  "kazakhstan" OR  "kazakh" OR  "kenya" OR  "democratic people’s republic of korea" OR  "north korea" OR  "korea" OR  "kosovo" OR  "kyrgyzstan" OR  "kirghizia" OR  "kirgizstan" OR  "kyrgyz republic" OR  "kirghiz" OR  "laos" OR  "lao pdr" OR  "lao people's democratic republic" OR  "lebanon" OR  "lebanese republic" OR  "lesotho" OR  "basutoland" OR  "liberia" OR  "libya" OR  "libyan arab jamahiriya" OR  "republic of north macedonia" OR  "macedonia" OR  "madagascar" OR  "malagasy republic" OR  "malawi" OR  "nyasaland" OR  "malaysia" OR  "malay federation" OR  "malaya federation" OR  "maldives" OR  "indian ocean islands" OR  "mali" OR  "micronesia" OR  "federated states of micronesia" OR  "kiribati" OR  "marshall islands" OR  "nauru" OR  "northern mariana islands" OR  "palau" OR  "tuvalu" OR  "mauritania" OR  "mauritius" OR  "mexico" OR  "moldova" OR  "moldovian" OR  "mongolia" OR  "montenegro" OR  "morocco" OR  "ifni" OR  "mozambique" OR  "portuguese east africa" OR  "myanmar" OR  "burma" OR  "namibia" OR  "nepal" OR  "netherlands antilles" OR  "nicaragua" OR  "niger" OR  "nigeria" OR  "muscat" OR  "pakistan" OR  "papua new guinea" OR  "new guinea" OR  "paraguay" OR  "peru" OR  "philippines" OR  "philipines" OR  "phillipines" OR  "phillippines" OR  "rwanda" OR  "ruanda" OR  "samoa" OR  "pacific islands" OR  "polynesia" OR  "samoan islands" OR  "navigator island" OR  "navigator islands" OR  "sao tome and principe" OR  "senegal" OR  "serbia" OR  "sierra leone" OR  "melanesia" OR  "solomon island" OR  "solomon islands" OR  "norfolk island" OR  "norfolk islands" OR  "somalia" OR  "south africa" OR  "south sudan" OR  "sri lanka" OR  "ceylon" OR  "saint lucia" OR  "st. lucia" OR  "saint vincent and the grenadines" OR  "saint vincent" OR  "st. vincent" OR  "grenadines" OR  "sudan" OR  "suriname" OR  "surinam" OR  "dutch guiana" OR  "netherlands guiana" OR  "syria" OR  "syrian arab republic" OR  "tajikistan" OR  "tadjikistan" OR  "tadzhikistan" OR  "tadzhik" OR  "tanzania" OR  "tanganyika" OR  "thailand" OR  "siam" OR  "timor leste" OR  "east timor" OR  "togo" OR  "togolese republic" OR  "tonga" OR  "tunisia" OR  "turkey" OR  "turkmenistan" OR  "turkmen" OR  "uganda" OR  "ukraine" OR  "uzbekistan" OR  "uzbek" OR  "vanuatu" OR  "new hebrides" OR  "vietnam" OR  "viet nam" OR  "middle east" OR  "west bank" OR  "gaza" OR  "palestine" OR  "yemen" OR  "zambia" OR  "zimbabwe" OR  "northern rhodesia" OR  "global south" OR  "africa south of the sahara" OR  "sub-saharan africa" OR  "subsaharan africa" OR  "central africa" OR  "north africa" OR  "northern africa" OR  "magreb" OR  "maghrib" OR  "sahara" OR  "southern africa" OR  "east africa" OR  "eastern africa" OR  "west africa" OR  "western africa" OR  "west indies" OR  "caribbean" OR  "central america" OR  "latin america" OR  "south and central america" OR  "south america" OR  "central asia" OR  "north asia" OR  "northern asia" OR  "southeastern asia" OR  "south eastern asia" OR  "southeast asia" OR  "south east asia" OR  "western asia" OR  "east europe" OR  "eastern europe" OR  "developing country" OR  "developing countries" OR  "developing nation?" OR  "developing population?" OR  "developing world" OR  "less developed countr*" OR  "less developed nation?" OR  "less developed population?" OR  "less developed world" OR  "lesser developed countr*" OR  "lesser developed nation?" OR  "lesser developed population?" OR  "lesser developed world" OR  "under developed countr*" OR  "under developed nation?" OR  "under developed population?" OR  "under developed world" OR  "underdeveloped countr*" OR  "underdeveloped nation?" OR  "underdeveloped population?" OR  "underdeveloped world" OR  "middle income countr*" OR  "middle income nation?" OR  "middle income population?" OR  "low income countr*" OR  "low income nation?" OR  "low income population?" OR  "lower income countr*" OR  "lower income nation?" OR  "lower income population?" OR  "underserved countr*" OR  "underserved nation?" OR  "underserved population?" OR  "underserved world" OR  "under served countr*" OR  "under served nation?" OR  "under served population?" OR  "under served world" OR  "deprived countr*" OR  "deprived nation?" OR  "deprived population?" OR  "deprived world" OR  "poor countr*" OR  "poor nation?" OR  "poor population?" OR  "poor world" OR  "poorer countr*" OR  "poorer nation?" OR  "poorer population?" OR  "poorer world" OR  "developing econom*" OR  "less developed econom*" OR  "lesser developed econom*" OR  "under developed econom*" OR  "underdeveloped econom*" OR  "middle income econom*" OR  "low income econom*" OR  "lower income econom*" OR  "low gdp" OR  "low gnp" OR  "low gross domestic" OR  "low gross national" OR  "lower gdp" OR  "lower gnp" OR  "lower gross domestic" OR  "lower gross national" OR  "lmic" OR  "lmics" OR  "third world" OR  "lami countr*" OR  "transitional countr*" OR  "emerging economies" OR  "emerging nation?") | 326,913 |
| **Combo** | #1 AND #2 AND #3 | 113 |
| **Limits** | AND English Language  AND DOP=2020-01-01/2024-12-31 |  |
| **Total** |  | 14 |

| Global Index Medicus | | |
| --- | --- | --- |
| **Date Searched** | 2/23/2026 | |
| **Concept** | **Search String** | **Results** |
| **1** | "climate chang*" OR  "climate warm*" OR  "warming climate" OR  "global warming" OR  "warm* temperature*" OR  "rising temperature*" OR  "extreme temperature*" OR  "extreme weather" OR  "extreme heat" OR  "rain*" OR  "flood*" OR  "urbanization" OR  "urbanisation" OR  "urban sprawl*" OR  "urban expan*" OR  "population grow*" | 4,923 |
| **2** | ("dengue" OR  "break bone fever" OR  "breakbone fever" OR  "Singapore hemorrhagic fever" OR  "Thai hemorrhagic fever" OR  "Philippine hemorrhagic fever") | 8,601 |
| **Combo** | 1 AND 2 | 175 |
| **Limits** | AND English Language  AND 2020-2024 |  |
| **Total** |  | 14 |
