# Supplementary material for: Battling dengue in a warming world: How climate and urbanization shape transmission in low- and middle-income countries (a rapid review)
Source: PLoS Negl Trop Dis. 2026 Jul 20;20(7):e0013758. doi: 10.1371/journal.pntd.0013758 (PMC13405114; doi:10.1371/journal.pntd.0013758)
Supplement: Attachment — Submitted filename: Rebuttal Letter (PNTD-D-25-02012R2).docx [file pntd.0013758.s008.docx]

**Responses to Reviewers**

**PNTD-D-25-02012R1: Battling Dengue in a Warming World: How Urbanization and Government Actions Impact Spread in Low- and Middle-Income Countries (A Rapid Review)**

Note: For the editor and reviewers’ convenience, all editor and reviewer comments are shown in blue, all quoted revisions in this response letter are highlighted in red, and all changes in the manuscript are shown using track changes. All line numbers referenced in our responses correspond to the revised manuscript with track changes.

**Reviewer’s Comments:**

Thank you again for the opportunity to review this work. The authors have properly addressed the majority of my concerns, and the manuscript has been significantly improved. However, before I can fully endorse the publication, there are two minor, yet methodologically critical, discrepancies between the rebuttal letter and the revised manuscript that must be resolved:

Dear Dr. Iuri Valerio Graciano Borges, we sincerely thank you for the time and effort you devoted to reviewing our manuscript again. In response to your comments, we have carefully revised the manuscript to reflect the changes. For your convenience, all changes have been clearly tracked in the revised manuscript.

SciELO and LILACS databases: In the "Response to Reviewers", the authors state they conducted an additional search in SciELO and LILACS via the Global Index Medicus to address regional bias. While I appreciate this effort, this supplementary search is not mentioned in the manuscript's Methods section and the PRISMA flow diagram. This additional search must be formally documented in the text, and the PRISMA flowchart must be updated to reflect the new numbers.

Thank you for highlighting this omission. We have now formally documented the supplementary search of the SciELO and LILACS databases, conducted through the Global Index Medicus, in Abstract (lines 71-72), the Methods section (line 186) and updated the search strategy (Supporting Information S6) accordingly.

Lines 71-72: “Six databases (MEDLINE, Embase, Global Health, SciELO, Global Index Medicus and Web of Science)...”

Line 186: “... SciELO (via Web of Science), and Global Index Medicus (all regional databases)...”

We have also revised the PRISMA flow diagram to include the records identified through these databases and to reflect the updated numbers throughout the screening and inclusion process.

Lines 238-240:

Justification for the 2020-2024 study period: The rebuttal letter provides a solid rationale for this specific 4-year window, explaining that it intentionally aligns with the "COVID-19 era" and its unique disruptions to health systems and vector control. However, this justification is entirely missing from the manuscript. A short timeframe for a climate change review requires explicit justification within the text so future readers understand the reasoning behind this methodological choice.

Thank you for this important comment. We have now incorporated the justification that we provided in the previous rebuttal letter into the revised manuscript in Lines 254–259. We explicitly clarified that the 2020–2024 study period was intentionally selected to align with the COVID-19 era and its distinctive disruptions to healthcare systems, surveillance capacity, mobility patterns, and vector control programs.

Lines 254-259: “The included studies were published between 2020 and 2024, indicating the recent and growing interest in dengue-related research under changing climatic and urban conditions. This time window was intentionally selected to align with the COVID-19 era, during which significant disruptions occurred in healthcare systems, surveillance capacity, and urban mobility patterns. Rather than providing a comprehensive historical synthesis of climate–dengue relationships, this review aimed to examine recent evidence generated within this distinct global context.”

We greatly appreciate your insightful and thoughtful comments, which have substantially improved the quality of our manuscript.
