# Supplementary material for: Battling dengue in a warming world: How climate and urbanization shape transmission in low- and middle-income countries (a rapid review)
Source: PLoS Negl Trop Dis. 2026 Jul 20;20(7):e0013758. doi: 10.1371/journal.pntd.0013758 (PMC13405114; doi:10.1371/journal.pntd.0013758)
Supplement: Attachment — Submitted filename: Responses to Reviewers (PNTD-D-25-02012).docx [file pntd.0013758.s007.docx]

**Responses to Reviewers**

**(PNTD-D-25-02012) Battling Dengue in a Warming World: How Climate and Urbanization Shape Transmission in Low- and Middle-Income Countries (A Rapid Review)**

Note: For the editor and reviewers’ convenience, all editor and reviewer comments are shown in blue, and all changes in the manuscript are shown using track changes. All line numbers referenced in our responses correspond to the revised manuscript with track changes.

**Editor’s Comments:**

Thank you for submitting your manuscript to PLOS Neglected Tropical Diseases. After careful consideration, we feel that it has merit but does not fully meet PLOS Neglected Tropical Diseases's publication criteria as it currently stands. Therefore, we invite you to submit a revised version of the manuscript that addresses the points raised during the review process.

Dear Dr. Josh M. Colston and reviewers, we sincerely thank you for the time and effort you devoted to reviewing our manuscript. We greatly appreciate your thoughtful comments, constructive suggestions, and encouraging feedback. In response to your insightful remarks, we have carefully revised the manuscript and believe that these revisions have substantially strengthened the scientific rigor and overall quality to ensure it meets publication criteria. For your convenience, all changes have been clearly tracked in the revised manuscript.

Additional Editor Comments: Although reviewer 1 recommends only minor revision, reviewer 2 raises serious concerns about the representativeness of the final set of included studies, that may require additional identification and screening of sources to address.

We thank the reviewers for highlighting this important concern. In response to Reviewer 2’s comments regarding the representativeness of the final set of included studies, we have thoroughly revised the manuscript and carefully re-evaluated the selection and screening process. Relevant clarifications and edits have been incorporated throughout the manuscript to strengthen transparency and justification of study inclusion. We believe that these revisions adequately address the concern raised and that the revised manuscript is now fully aligned with the journal’s standards.

**Reviewer’s Comments:**

Reviewer #1

**Methods**

The research question ("How are rising global temperatures and urbanization affecting the transmission season and geographic spread of dengue fever in LMICs, and how are local governments responding to these challenges?") currently does not precisely reflect what was investigated in this manuscript and needs some adjustment. First of all, the authors were not only interested in the effect of increasing temperatures and urbanization on transmission of dengue, but also other climate variables. This was inconsistently described in the manuscript. In Table 1, the focus area is said to be research/reports addressing effects of rising global temperatures and urbanization on dengue. However, in line 148, the authors wrote “Our focus was on dengue fever and climate change”. Additionally, the importance of rainfall, El Niño/La Niña, and humidity were highlighted in the introduction, but not mentioned in the methods. This made it confusing to know which variables were really of interest. Only in the results section, it became clear that the authors were not solely interested in temperature as a climate variable, but also rainfall and humidity. The authors should adjust the research question and methodology to reflect this. (ie How are rising global temperatures and urbanization affecting transmission → How are climate variables and urbanization affecting transmission).

We thank the reviewer for highlighting this important issue regarding clarity and consistency. We have revised the research question (lines 140-142), eligibility criteria (lines 157-158), and Table 1 (line 169) to explicitly reflect that our review examined multiple climate variables including temperature, rainfall, and humidity. We also clarified in the Methods section that these climate variables were intentionally extracted and synthesized. These revisions align the stated objectives and methodology with the analyses presented in the Results and improve transparency regarding the scope of climate factors considered.

Second, while the research question specifically focuses on effects on transmission season and geographic spread of dengue, the study discusses the effect on dengue transmission more broadly. For example, the results section mentions impacts such as ‘increased risk’ and ‘localized hotspots’ as well as the lengthening transmission season and expansion into higher altitude areas. It is great that the authors mention these impacts as well, however, rephrasing the first part of the RQ may better reflect the study: “How are climate variables and urbanization affecting the transmission of dengue virus in LMICs...?”

We thank the reviewer for this suggestion. We agree that while the original research question emphasized transmission season and geographic spread, our synthesis also examined broader impacts on dengue transmission, including increased risk, localized hotspots, and urban environmental effects. We have therefore revised the research question to more accurately reflect the scope of the review.

**Results**

Most of the results are presented clearly and succinctly. However, the geographic distribution of included studies should be reported more clearly:

2.1 In line 222-224, the authors write “All studies were conducted in low- and middle-income countries, with India, the Philippines, and Brazil among the most frequently represented countries.” However, in S1 Table 2, none of the studies were reportedly conducted in Brazil, while many studies were conducted in Bangladesh. The authors should revise lines 222-224 for accuracy.

Thank you for highlighting this inconsistency. We agree with the reviewer that the original wording was inaccurate. We have revised lines 243–245 to remove Brazil and to accurately reflect the study settings reported in S1 Table 2.

2.2 I suggest including a map as a figure to show the geographic distribution of the included studies, with a colour gradient to represent the number of studies per country. This would make it instantly clear that almost all the included studies were focused on South and Southeast Asia, with very few studies focusing on countries outside those regions.

We thank the reviewer for this helpful suggestion. In response, we have added Figure 2 (line 249) presenting a map of the geographic distribution of the included studies, with a color gradient indicating the number of studies per country.

2.3 Is the geographical distribution of included studies representative of the global distribution of the burden of dengue? Could refer to Cattarino et al. 2020 (<https://doi.org/10.1126/scitranslmed.aax4144>) or Yang et al. 2021 (<https://doi.org/10.1093/jtm/taab146> ) for this. The authors should reflect on why the distribution of included studies is the way it is, and how it may influence the results. It could be a major limitation to have so few studies in other areas of the world strongly affected by dengue, like South America.

We appreciate the reviewer’s important point regarding the representativeness of the included studies. We have added a reflection in the Limitations section (lines 383-388) acknowledging that the geographic distribution of studies does not fully mirror the global burden of dengue, which is substantial in Latin America and parts of sub-Saharan Africa.

**Conclusions**

The authors make some claims in the conclusions that extend beyond what the results can support. For example, the results report that rainfall was generally reported to be associated with increased dengue risk, while the conclusion claims that "shifting rainfall patterns" associated with climate change are altering transmission patterns, which is not directly supported by the results. Associations between rainfall and dengue risk could potentially simply reflect seasonal variability in risk, rather than long-term climate change. It would be beneficial to more clearly distinguish between short-term weather-dengue associations and the broader, long-term impacts of climate change. Discussing climate change is definitely relevant and warranted, but it would help to be more precise about the distinction between the empirical evidence from the results (ie associations with short-term weather) and the potential implications under future climate change (ie expected long-term changes).

Thank you for this important comment. We have revised the conclusion to more precisely reflect the evidence synthesized in this review. Specifically, we now describe higher temperatures and rainfall variability as factors commonly associated with dengue transmission dynamics, while framing climate change as a relevant broader context and potential future influence rather than a direct empirical finding of our results.

The lack of geographic distribution across regions affected by dengue should be considered as a limitation.

Thank you for this observation. Given the limitations outlined in our discussion, the geographic distribution of studies reflects the way the sources were analyzed and the criteria used to classify countries as low- and middle-income countries. As a result, the studies included in this review represent the countries that met these criteria within our search strategy. If the review had been broader in scope, we would have included additional countries and regions affected by dengue.

**General Comments**

Line 142-143: “This study was determined not to involve human subjects” can be simplified to “This study does not involve human subjects”

Thank you for the advice. We have made the revision (lines 159-150)*.*

Paragraph 152-156 is repeating 142-146

Thank you for the observation, we have removed the repetitive content.

**Summary**

A key strength of this manuscript is the consideration of climate variables, urbanization and government responses simultaneously. This approach is valuable as it moves beyond isolated risk factors, and captures the various interconnected factors that influence dengue transmission, while also providing insight into potential adaptation strategies.

Thank you for this thoughtful comment. We appreciate the recognition of the manuscript’s integrated approach. Our intention in examining climate variables, urbanization, and government responses simultaneously was to capture the multifactorial and interconnected drivers of dengue transmission rather than treating these factors in isolation.

Minor comments:

1. Additional details about breeding and biting behaviour on Aedes aegypti.

In lines 100-101, the authors write: “Climate variability plays an important role in modulating mosquito breeding and virus transmission.” I suggest adding more details about the breeding and biting behaviour Aedes aegypti specifically (container-breeding, diurnal & peak biting times, indoor/outdoor biting). These provide crucial context to understand the links to urbanization and climate variables, and what vector control strategies are relevant. (Rund et al. 2020 found that artificial light (ie from urbanization) can increase night time biting of Aedes aegypti for example: <https://doi.org/10.4269/ajtmh.20-0885>)

We appreciate this insightful suggestion. We have revised the Introduction (lines 100-106) to strengthen the biological context linking climate variability to dengue transmission, including the effects of temperature on mosquito development, biting frequency, and viral replication. We agree that additional vector-specific behavioral detail may be informative; however, to maintain the concision and focus of the Introduction, we limited the revision to the aspects most directly relevant to the climate–transmission relationship examined in this review.

2. In lines 119-121 the authors mention that there is limited diagnostic access in endemic African countries. I suggest to explain here or earlier in the introduction how dengue is usually diagnosed in LMICs.

Thank you for this helpful suggestion. We have added content (lines 126-127) to clarify how dengue is typically diagnosed in LMICs.

3. Parallels with Malaria Surveillance and Control

In line 107-113, the authors describe the challenges in dengue control in LMICs, such as resource constraints, fragmented surveillance, limited public awareness, lack of integration into health system, etc. And in line 338-342, the authors write: “Although some governments have introduced promising strategies such as early warning systems and risk mapping tools, these efforts are frequently limited in scope, poorly coordinated, or lacking sustainable funding support [32, 41, 67, 75]. There remains limited documentation of full-scale implementation into routine public health planning.”

I suggest to mention in paragraph 107-113 in the introduction that efforts to control malaria have often been stronger, with larger scope, better coordination, better funding, and have also been integrated into national health systems. I suggest discussing how this work on malaria can be leveraged to battle dengue. For example, integrating dengue into already existing national Malaria Surveillance Systems that incorporate temperature and rainfall data to increase preparedness. I suggest highlighting the value of a multi-disease approach, preventing parallel single-disease systems or strategies, especially relevant for VBDs as many vector control strategies tackle multiple VBDs simultaneously (ie Integrated Vector Management, WHO recommended approach).

Thank you for the thought suggestion. We agree that existing malaria surveillance and control platforms may offer useful lessons for broader vector-borne disease preparedness and integrated response strategies in some settings. However, because this review was specifically designed to examine dengue-related evidence, and our included studies did not assess malaria programs or cross-disease surveillance integration, we were cautious about introducing this comparison into the manuscript. Therefore, we chose not to expand the text in this direction in order to maintain the dengue-specific scope and avoid implying findings beyond the evidence synthesized in our review.

4. In line 126-127 “Vulnerability varies by population, with age, location, and urban setting influencing infection risk.” I suggest being more specific here. Which ages, locations, and urban settings are more at risk?

We appreciate this helpful suggestion. We have revised the sentence (lines 132-134) to state higher dengue incidence among people aged 15 years and older and in central, more densely built-up urban areas compared with children and residents of peripheral areas.

5. The authors may want to mention the new dengue vaccine, TAK-003, in the discussion, as a new potential government response, recommended by WHO

Thank you for the advice. We have added a brief statement with a new reference to the Discussion (lines 374-376) acknowledging that, although vaccination was not addressed in our included studies, tools such as the TAK-003 dengue vaccine may become relevant to future governmental response strategies in high-burden settings. Dear reviewer, thank you very much for the time and effort in providing us encouraging feedback and constructive advice.

Reviewer #2

**Methods**

The study design presents significant limitations that hinder its ability to fully address the stated objectives. First, the decision to conduct a "rapid review" rather than a full systematic review is questionable for a chronic, endemic issue like dengue, as it lacks the necessary depth to capture complex long-term trends. Consequently, the selected time window (2020–2024) is insufficient to adequately assess climate change impacts, as it discards valuable longitudinal data published prior to this period. Furthermore, limiting the search to English-language articles and excluding key regional databases (such as LILACS or SciELO) introduces a severe selection bias. This effectively excludes a vast amount of relevant literature from Latin America, which is critical for any study focusing on LMICs. Regarding the study population, there is a major factual discrepancy: the text claims Brazil is one of the most represented countries, yet the provided supplementary tables contain zero entries for Brazil, rendering the population description inaccurate. Finally, the presence of untreated duplicates in the reference list raises concerns about the accuracy of the final sample size (i.e., n=65) and the validity of the statistical synthesis derived from it.

Thank you for the insightful comments regarding our search strategy and database coverage. In response, we conducted an additional search to strengthen the comprehensiveness of the review. To address concerns about regional representation, we added SciELO and LILACS via the Global Index Medicus to our database search. To maintain consistency with the original COVID-era framing of the rapid review, the additional searches were limited to the same time window (2020–2024) used in the initial search strategy. The searches from SciELO and Global Index Medicus produced 10 unique records after deduplication, which underwent additional title and abstract screening in Covidence. One article was eligible for full-text screening but excluded from further analysis because its study period fell outside our inclusion criteria. We also conducted an additional deduplication in the reference list to ensure that the final screening pool reflects only unique references.

**Results**

The results presented in the manuscript are neither completely accurate nor consistent with the analysis plan implied by the study's title. There is a critical contradiction where the text cites Brazil as one of the most frequently represented countries, yet a thorough check of Supplementary Tables S1 and S2 reveals absolutely no entries for Brazil. This implies that the results section discusses data not present in the extraction tables, which significantly undermines the reliability of the findings.

Thank you for observing this inconsistency. As addressed in our response to the previous reviewer, we have revised relevant content (lines 243–245) accordingly to remove Brazil and to accurately reflect the study settings reported in S1 Table 2.

Furthermore, the quantitative synthesis is questionable due to data integrity issues; since the reference list contains untreated duplicates, it is unclear whether these were counted as separate data points in the final sample size. If these duplicates were included in the denominator, the reported percentages are statistically invalid.

Thank you for raising this concern. We would like to clarify that the duplicate entries identified in the reference list were editorial duplicates only and were not part of the screened or analyzed dataset. Prior to screening, all records were deduplicated in Covidence, and the screening statistics and final sample size were derived from that deduplicated set. Therefore, these duplicate reference entries did not affect the denominator or the reported percentages. However, we confirmed that the duplicate references led to some incorrect citation numbering in the manuscript. We have now corrected the reference list and updated the citation numbering accordingly.

Finally, the results do not align well with the title's promise of analyzing "government actions," as the authors admit this was a minor theme (found in only 29% of studies) compared to the heavy focus on climate.

We appreciate the reviewer’s observation. While it is correct that government responses were reported in 29% of the included studies, we believe it remains appropriate to retain this element in the title. Our research question explicitly aimed to examine not only how climate change and urbanization influence dengue transmission in LMICs, but also how governments are responding to these emerging challenges.

**Conclusions**

The conclusions drawn in the manuscript are not fully supported by the data presented. The most significant issue is the assertion that Brazil is a key represented country, a conclusion that is physically impossible to support given that the supplementary data contains zero Brazilian studies. This fundamental disconnect between the "evidence" (tables) and the "claims" (text) invalidates the conclusions regarding geographic distribution and representation.

Thank you for the comment. We agree that the earlier mention of Brazil was incorrect and have corrected this textual error in the revised manuscript. This issue resulted from imprecise wording in the narrative text rather than from an inconsistency in the underlying dataset, which is accurately reflected in the supplementary materials.

Additionally, while the authors briefly mention language bias as a limitation, they fail to adequately describe the magnitude of this limitation. By excluding Spanish and Portuguese literature, the review likely missed a substantial portion of relevant data on dengue in Latin America, skewing the conclusions about "Global" or "LMIC" trends.

Thank you for the observation on this limitation. To address this, we expanded the limitations section (lines 390-400) to state more explicitly that the included studies were unevenly distributed geographically and that this pattern may reflect structural differences in research funding, data availability, and publication output rather than true differences in dengue burden. We also now note more clearly that excluding non-English literature may have contributed to underrepresentation of relevant studies from Spanish- and Portuguese-speaking settings

The authors discuss public health relevance and government actions, but these conclusions are weakened by the fact that only 29% of their included studies actually documented government responses, making the strong focus on this aspect in the conclusion (and title) disproportionate to the actual findings.

While it is correct that government responses were documented in 29% of the included studies, our intention was not to suggest that this theme dominated the literature, but rather to synthesize the available evidence. The relatively limited number of studies examining governmental actions is itself an important finding, as it underscores a gap between the well-documented environmental drivers of dengue transmission and the comparatively limited evidence on how governments are responding to these challenges. For this reason, we believe it remains appropriate to retain discussion of government responses in both the title and conclusion. However, we have revised the discussion (lines 383-388) and the title to more clearly reflect the proportion of studies addressing this theme and to frame government responses as an emerging but underrepresented area in the literature.

**General Comments**

Line 8: The species name Aedes aegypti must be always italicized.

Thank you, we have correctly italicized the term.

Line 94: The term "tropic" is incorrect in this context; it should be "tropical" (Neglected Tropical Diseases).

Thank you for capturing that, we have updated the term in our keywords list.

Reference List: Reference 72 contains visible software coding tags (), which disrupt the data presentation and must be cleaned.

Thank you, we have revised Reference 72.

Line 85 (Abstract): The phrase "though many were interfered by" is non-standard and awkward. I suggest rewriting it as "though many were hindered by" or "hampered by" to enhance clarity and flow.

Thank you for the advice, we have made the revision.

Lines 152–156: This paragraph is a verbatim repetition of the text found in the "Study Design" section. It adds no value and should be deleted to improve the manuscript's conciseness.

We have removed the repetition, thank you.

Summary

This manuscript addresses a topic of high relevance: the intersection of climate change, urbanization, and dengue transmission in LMICs. However, the study suffers from significant methodological flaws and data inconsistencies that prevent its publication in its current form.

We acknowledge the concerns regarding the methodological limitations and data inconsistencies identified in the manuscript. These issues have been carefully addressed in the revised version through clarification of the methods, improved consistency in data reporting, and additional revisions to strengthen the analysis.

I observed that the supplementary tables (S1 and S2) correctly list 65 studies. However, the final Reference list contains untreated duplicates (ref. 71 identical to 73; ref. 75 identical to 79; ref. 76 identical to 81) and software formatting errors (ref. 72).

Thank you for the comment, we have updated the Reference list to remove all the duplicates.

Furthermore, I reinforce a serious inconsistency: the text claims that Brazil is one of the most represented countries, but there are no Brazilian studies listed in tables S1 and S2 or in the bibliography. This absence directly contradicts the statement that the country is one of the most represented. This suggests that either the supplementary data are incomplete, or the textual conclusion was based on studies that were not included in the final analysis. Please verify this.

Thank you for reinforcing this inconsistency, as addressed in our previous response, we have revised relevant content (lines 243–245) to remove Brazil and to accurately reflect the study settings reported.

I also have a concern regarding the title versus the content. The title highlights "Government Actions", but your results admit that only 29% of the studies documented this. It seems the paper promises more on government strategies than it actually delivers; the focus is heavily skewed towards climate (65%). Additionally, I question the choice of a "rapid review" for an endemic problem like Dengue. A full systematic review would have been more appropriate to provide the necessary depth.

We thank the reviewer for this comment. We have revised the title. As addressed above, although government responses were reported in 29% of the included studies, their inclusion in the content reflects one of the objectives of the review which is to examine both environmental drivers of dengue transmission and how governments are responding. The limited representation of governmental actions is itself an important finding and highlights a gap in the literature, which we now clarify in the Government Response subsection (lines 333-336) and discussion (lines 388-391). Regarding the use of a rapid review, this approach was selected to provide a timely synthesis of recent evidence (2020–2024) on climate change, urbanization, and dengue transmission in LMICs

Below are the errors I verified and my comments line by line:

- Line 8: Aedes aegypti must always be in italics.

Thank you, we have updated this.

- Line 85 and Abstract: In the phrase "though many were interfered by", the phrasing sounds strange; rewrite using another term like "hindered" which fits the context better.

We have revised this, thank you.

- Line 94: "tropic" should be "tropical".

We have updated this.

- Line 134 (Introduction): I felt the lack of a paragraph speaking about the role of temperature on the vector and dengue transmission. How does it affect it? Only the role of El Niño in Central America was mentioned, but that is all. You could report the regulatory and amplifying role that temperature has, that high and low temperatures naturally regulate cases, and that with the increase in global average temperatures, we could have both an increase in dengue occurrence regions and a decrease in hotter regions. Saying this is important as it links directly with the point of the article which is climate change and the increase in global temperatures. El Niño is important, but it is a climate modulator, not an effect of climate change.

Thank you for this insightful suggestion. We agree that the role of temperature in dengue transmission warranted clearer explanation beyond the discussion of El Niño. We have revised the Introduction (lines 100-108) to describe how temperature influences mosquito development, biting frequency, and viral replication within the vector. We also clarified that this relationship is nonlinear, such that both low and very high temperatures can constrain transmission, and that rising temperatures may increase dengue suitability in some regions while reducing it in others that become too hot for efficient transmission.

- Lines 152 to 156: This entire paragraph is repeating from the "Study Design", please exclude it.

We have addressed this issue, thank you.

- Methods (Time Window): The study only covers 2020 to 2024. For a paper on climate change, a 4-year window seems extremely short. By doing this, you are discarding important longitudinal studies published before 2020 that are crucial for understanding these trends.

The 2020–2024 time window was intentionally selected to align with the COVID-19 era, during which significant disruptions in healthcare systems, surveillance capacity, urban mobility patterns, and vector control programs occurred. Our goal was not to conduct a comprehensive historical synthesis of climate–dengue relationships, but rather to examine recent evidence emerging during this specific global context, where environmental, social, and health system pressures intersected in unique ways.

- Table 1, Language row: This may have limited the results quite a lot. Of the 65 articles selected in the end, much more could have been obtained if documents in Spanish and/or Portuguese had been included. You missed important regional databases like LILACS or SciELO. Excluding these introduces a severe geographical bias, as Latin American science on Dengue is often published in these languages.

Thank you for highlighting this important concern regarding potential geographic and language bias. To address this issue, we expanded our database search to include SciELO and LILACS through the Global Index Medicus, as these databases capture a substantial portion of Latin American scientific literature that may not be indexed in the databases originally searched. While this additional search was conducted to improve regional representation, it yielded only 10 additional records for screening and did not substantially change the overall findings or conclusions of the review.

- Line 215: Most studies are from South and Southeast Asia; as I mentioned, the limitation of the document being in English and the exclusion of regional databases may have limited the results a lot. Not including many studies from Lusophone and Hispanophone countries that are constantly in contact with dengue in Africa and the Americas.

Thank you for this important observation. We agree that limiting the search to English-language publications and initially excluding some regional databases may have reduced the representation of studies from Lusophone and Hispanophone countries where dengue is also highly prevalent. However, expanding the linguistic scope of the review was beyond the methodological scope and resources of this rapid review.

- Line 223 on "Brazil": I checked the two tables mentioned so far and did not find any mention of Brazil. Revise.

Thank you, as addressed in our previous response, we have revised relevant content (lines 243–245) to remove Brazil and to accurately reflect the study settings reported.

- Lines 255 to 258: For this very reason, I believe a paragraph describing the possible effects of temperature should be included in the introduction.

Thank you for the advice. The introduction has been updated to include discussion of the potential effects of temperature and rainfall on dengue transmission (Lines 100–106).

- Line 356: Good that the language bias was mentioned, it really affected the representativeness of the data.

Thank you for this comment, we appreciate having included the language bias as well.

Besides this, I also observed duplicates in the list of references:

- Reference 71 identical to 73

- Reference 75 identical to 79

- Reference 76 identical to 81

We have updated the references to remove duplicates. We appreciate the reviewer for all the thoughtful comments, as well as the time and effort you have contributed to evaluate our manuscript.
