# Supplementary material for: Battling dengue in a warming world: How climate and urbanization shape transmission in low- and middle-income countries (a rapid review)
Source: PLoS Negl Trop Dis. 2026 Jul 20;20(7):e0013758. doi: 10.1371/journal.pntd.0013758 (PMC13405114; doi:10.1371/journal.pntd.0013758)
Supplement: Peer review history

# Peer review: Decision Letter 0

**DOI:** 10.1371/journal.pntd.0013758.r001

**Authors:** Josh M Colston, David Safronetz

26 Jan 2026

PNTD-D-25-02012

Battling Dengue in a Warming World: How Urbanization and Government Actions Impact Spread in Low- and Middle-Income Countries (A Rapid Review)

PLOS Neglected Tropical Diseases

Dear Dr. Yu,

Thank you for submitting your manuscript to PLOS Neglected Tropical Diseases. After careful consideration, we feel that it has merit but does not fully meet PLOS Neglected Tropical Diseases's publication criteria as it currently stands. Therefore, we invite you to submit a revised version of the manuscript that addresses the points raised during the review process.

Please submit your revised manuscript within by Mar 27 2026 11:59PM. If you will need more time than this to complete your revisions, please reply to this message or contact the journal office at plosntds@plos.org. When you're ready to submit your revision, log on to https://www.editorialmanager.com/pntd/ and select the 'Submissions Needing Revision' folder to locate your manuscript file.

Please include the following items when submitting your revised manuscript:

* A letter that responds to each point raised by the editor and reviewer(s). You should upload this letter as a separate file labeled 'Response to Reviewers'. This file does not need to include responses to any formatting updates and technical items listed in the 'Journal Requirements' section below.

* A marked-up copy of your manuscript that highlights changes made to the original version. You should upload this as a separate file labeled 'Revised Manuscript with Track Changes'.

* An unmarked version of your revised paper without tracked changes. You should upload this as a separate file labeled 'Manuscript'.

If you would like to make changes to your financial disclosure, competing interests statement, or data availability statement, please make these updates within the submission form at the time of resubmission. Guidelines for resubmitting your figure files are available below the reviewer comments at the end of this letter.

We look forward to receiving your revised manuscript.

Kind regards,

Josh M Colston, Ph.D.

Academic Editor

PLOS Neglected Tropical Diseases

David Safronetz

Section Editor

PLOS Neglected Tropical Diseases

Shaden Kamhawi

co-Editor-in-Chief

PLOS Neglected Tropical Diseases

orcid.org/0000-0003-4304-636XX

Paul Brindley

co-Editor-in-Chief

PLOS Neglected Tropical Diseases

orcid.org/0000-0003-1765-0002

**Additional Editor Comments:**

Although reviewer 1 recommends only minor revision, reviewer 2 raises serious concerns about the representativeness of the final set of included studies, that may require additional identification and screening of sources to address.

**Journal Requirements:**

1) Please provide an Author Summary. This should appear in your manuscript between the Abstract (if applicable) and the Introduction, and should be 150-200 words long. The aim should be to make your findings accessible to a wide audience that includes both scientists and non-scientists. Sample summaries can be found on our website under Submission Guidelines:

https://journals.plos.org/plosntds/s/submission-guidelines#loc-parts-of-a-submission

2) Please upload all main figures as separate Figure files in .tif or .eps format. For more information about how to convert and format your figure files please see our guidelines:

https://journals.plos.org/plosntds/s/figures

**Reviewers' Comments:**

Reviewer's Responses to Questions

**Key Review Criteria Required for Acceptance?**

As you describe the new analyses required for acceptance, please consider the following:

**Methods**

-Are the objectives of the study clearly articulated with a clear testable hypothesis stated?

-Is the study design appropriate to address the stated objectives?

-Is the population clearly described and appropriate for the hypothesis being tested?

-Is the sample size sufficient to ensure adequate power to address the hypothesis being tested?

-Were correct statistical analysis used to support conclusions?

-Are there concerns about ethical or regulatory requirements being met?

Reviewer #1: 1. The research question ("How are rising global temperatures and urbanization affecting the transmission season and geographic spread of dengue fever in LMICs, and how are local governments responding to these challenges?") currently does not precisely reflect what was investigated in this manuscript and needs some adjustment.

First of all, the authors were not only interested in the effect of increasing temperatures and urbanization on transmission of dengue, but also other climate variables. This was inconsistently described in the manuscript. In Table 1, the focus area is said to be research/reports addressing effects of rising global temperatures and urbanization on dengue. However, in line 148, the authors wrote “Our focus was on dengue fever and climate change”. Additionally, the importance of rainfall, El Niño/La Niña, and humidity were highlighted in the introduction, but not mentioned in the methods. This made it confusing to know which variables were really of interest. Only in the results section, it became clear that the authors were not solely interested in temperature as a climate variable, but also rainfall and humidity. The authors should adjust the research question and methodology to reflect this. (ie How are rising global temperatures and urbanization affecting transmission → How are climate variables and urbanization affecting transmission).

Second, while the research question specifically focuses on effects on transmission season and geographic spread of dengue, the study discusses the effect on dengue transmission more broadly. For example, the results section mentions impacts such as ‘increased risk’ and ‘localized hotspots’ as well as the lengthening transmission season and expansion into higher altitude areas. It is great that the authors mention these impacts as well, however, rephrasing the first part of the RQ may better reflect the study: “How are climate variables and urbanization affecting the transmission of dengue virus in LMICs...?”

Reviewer #2: Are the objectives of the study clearly articulated with a clear testable hypothesis stated? Yes

Is the study design appropriate to address the stated objectives? No

Is the population clearly described and appropriate for the hypothesis being tested? No

Is the sample size sufficient to ensure adequate power to address the hypothesis being tested? No

Were correct statistical analysis used to support conclusions? No

Are there concerns about ethical or regulatory requirements being met? No

Bellow are the full details:

The study design presents significant limitations that hinder its ability to fully address the stated objectives. First, the decision to conduct a "rapid review" rather than a full systematic review is questionable for a chronc, endemic issue like dengue, as it lacks the necessary depth to capture complex long-term trends. Consequently, the selected time window (2020–2024) is insufficient to adequately assess climate change impacts, as it discards valuable longitudinal data published prior to this period. Furthermore, limiting the search to English-language articles and excluding key regional databases (such as LILACS or SciELO) introduces a severe selection bias. This effectively excludes a vast amount of relevant literature from Latin America, which is critical for any study focusing on LMICs. Regarding the study population, there is a major factual discrepancy: the text claims Brazil is one of the most represented countries, yet the provided supplementary tables contain zero entries for Brazil, rendering the population description inaccurate. Finally, the presence of untreated duplicates in the reference list raises concerns about the accuracy of the final sample size (i.e., n=65) and the validity of the statistical synthesis derived from it.

**********

**Results**

-Does the analysis presented match the analysis plan?

-Are the results clearly and completely presented?

-Are the figures (Tables, Images) of sufficient quality for clarity?

Reviewer #1: Most of the results are presented clearly and succinctly. However, the geographic distribution of included studies should be reported more clearly:

2.1 In line 222-224, the authors write “All studies were conducted in low- and middle-income countries, with India, the Philippines, and Brazil among the most frequently represented countries.” However, in S1 Table 2, none of the studies were reportedly conducted in Brazil, while many studies were conducted in Bangladesh. The authors should revise lines 222-224 for accuracy.

2.2 I suggest including a map as a figure to show the geographic distribution of the included studies, with a colour gradient to represent the number of studies per country. This would make it instantly clear that almost all the included studies were focused on South and Southeast Asia, with very few studies focusing on countries outside those regions.

2.3 Is the geographical distribution of included studies representative of the global distribution of the burden of dengue? Could refer to Cattarino et al. 2020 (https://doi.org/10.1126/scitranslmed.aax4144) or Yang et al. 2021 (https://doi.org/10.1093/jtm/taab146 ) for this. The authors should reflect on why the distribution of included studies is the way it is, and how it may influence the results. It could be a major limitation to have so few studies in other areas of the world strongly affected by dengue, like South America.

Reviewer #2: Does the analysis presented match the analysis plan? No

Are the results clearly and completely presented? No

Are the figures (Tables, Images) of sufficient quality for clarity? Yes (could be better resolution)

The results presented in the manuscript are neither completely accurate nor consistent with the analysis plan implied by the study's title. There is a critical contradiction where the text cites Brazil as one of the most frequently represented countries, yet a thorough check of Supplementary Tables S1 and S2 reveals absolutely no entries for Brazil. This implies that the results section discusses data not present in the extraction tables, which significantly undermines the reliability of the findings. Furthermore, the quantitative synthesis is questionable due to data integrity issues; since the reference list contains untreated duplicates, it is unclear whether these were counted as separate data points in the final sample size. If these duplicates were included in the denominator, the reported percentages are statistically invalid. Finally, the results do not align well with the title's promise of analyzing "government actions," as the authors admit this was a minor theme (found in only 29% of studies) compared to the heavy focus on climate.

**********

**Conclusions**

-Are the conclusions supported by the data presented?

-Are the limitations of analysis clearly described?

-Do the authors discuss how these data can be helpful to advance our understanding of the topic under study?

-Is public health relevance addressed?

Reviewer #1: 3.1 The authors make some claims in the conclusions that extend beyond what the results can support. For example, the results report that rainfall was generally reported to be associated with increased dengue risk, while the conclusion claims that "shifting rainfall patterns" associated with climate change are altering transmission patterns, which is not directly supported by the results. Associations between rainfall and dengue risk could potentially simply reflect seasonal variability in risk, rather than long-term climate change. It would be beneficial to more clearly distinguish between short-term weather-dengue associations and the broader, long-term impacts of climate change. Discussing climate change is definitely relevant and warranted, but it would help to be more precise about the distinction between the empirical evidence from the results (ie associations with short-term weather) and the potential implications under future climate change (ie expected long-term changes).

3.2 The lack of geographic distribution across regions affected by dengue should be considered as a limitation.

Reviewer #2: Are the conclusions supported by the data presented? No

Are the limitations of analysis clearly described? No

Do the authors discuss how these data can be helpful to advance our understanding of the topic under study? Yes

Is public health relevance addressed? Yes

The conclusions drawn in the manuscript are not fully supported by the data presented. The most significant issue is the assertion that Brazil is a key represented country, a conclusion that is physically impossible to support given that the supplementary data contains zero Brazilian studies. This fundamental disconnect between the "evidence" (tables) and the "claims" (text) invalidates the conclusions regarding geographic distribution and representation. Additionally, while the authors briefly mention language bias as a limitation, they fail to adequately describe the magnitude of this limitation. By excluding Spanish and Portuguese literature, the review likely missed a substantial portion of relevant data on dengue in Latin America, skewing the conclusions about "Global" or "LMIC" trends. The authors discuss public health relevance and government actions, but these conclusions are weakened by the fact that only 29% of their included studies actually documented government responses, making the strong focus on this aspect in the conclusion (and title) disproportionate to the actual findings.

**********

**Editorial and Data Presentation Modifications?**

Use this section for editorial suggestions as well as relatively minor modifications of existing data that would enhance clarity. If the only modifications needed are minor and/or editorial, you may wish to recommend “Minor Revision” or “Accept”.

Reviewer #1: 4.1 Line 142-143: “This study was determined not to involve human subjects” can be simplified to “This study does not involve human subjects”

4.2 Paragraph 152-156 is repeating 142-146

Reviewer #2: Line 8: The species name Aedes aegypti must be always italicized.

Line 94: The term "tropic" is incorrect in this context; it should be "tropical" (Neglected Tropical Diseases).

Reference List: Reference 72 contains visible software coding tags (<ovid:i>), which disrupt the data presentation and must be cleaned.

Line 85 (Abstract): The phrase "though many were interfered by" is non-standard and awkward. I suggest rewriting it as "though many were hindered by" or "hampered by" to enhance clarity and flow.

Lines 152–156: This paragraph is a verbatim repetition of the text found in the "Study Design" section. It adds no value and should be deleted to improve the manuscript's conciseness.</ovid:i>

**********

**Summary and General Comments**

Use this section to provide overall comments, discuss strengths/weaknesses of the study, novelty, significance, general execution and scholarship. You may also include additional comments for the author, including concerns about dual publication, research ethics, or publication ethics. If requesting major revision, please articulate the new experiments that are needed.

Reviewer #1: Summary

This study investigated the influence of climate variables and urbanization on the transmission of dengue virus in LMICs and government responses to these challenges through a rapid systematic review of empirical studies. Among the 65 included studies, 42 found positive associations between rising temperatures and dengue transmission, and two found non-linear relationships with temperature. Rainfall was generally also found to be positively associated with increased dengue risk, although some studies found mixed results that were dependent on rainfall intensity, infrastructure, and local water storage practices. Humidity was less commonly investigated in the included literature, but found to increase mosquito activity, particularly at high temperatures. Urbanization, investigated in 23 of the studies, was found to significantly influence dengue transmission in LMICs due to factors such as informal settlements, population density, inadequate WaSH, and land-use change.

A key strength of this manuscript is the consideration of climate variables, urbanization and government responses simultaneously. This approach is valuable as it moves beyond isolated risk factors, and captures the various interconnected factors that influence dengue transmission, while also providing insight into potential adaptation strategies.

Minor comments:

1. Additional details about breeding and biting behaviour on Aedes aegypti.

In lines 100-101, the authors write: “Climate variability plays an important role in modulating mosquito breeding and virus transmission.” I suggest adding more details about the breeding and biting behaviour Aedes aegypti specifically (container-breeding, diurnal & peak biting times, indoor/outdoor biting). These provide crucial context to understand the links to urbanization and climate variables, and what vector control strategies are relevant. (Rund et al. 2020 found that artificial light (ie from urbanization) can increase night time biting of Aedes aegypti for example: https://doi.org/10.4269/ajtmh.20-0885)

2. In lines 119-121 the authors mention that there is limited diagnostic access in endemic African countries. I suggest to explain here or earlier in the introduction how dengue is usually diagnosed in LMICs.

3. Parallels with Malaria Surveillance and Control

In line 107-113, the authors describe the challenges in dengue control in LMICs, such as resource constraints, fragmented surveillance, limited public awareness, lack of integration into health system, etc. And in line 338-342, the authors write: “Although some governments have introduced promising strategies such as early warning systems and risk mapping tools, these efforts are frequently limited in scope, poorly coordinated, or lacking sustainable funding support [32, 41, 67, 75]. There remains limited documentation of full-scale implementation into routine public health planning.”

I suggest to mention in paragraph 107-113 in the introduction that efforts to control malaria have often been stronger, with larger scope, better coordination, better funding, and have also been integrated into national health systems. I suggest discussing how this work on malaria can be leveraged to battle dengue. For example, integrating dengue into already existing national Malaria Surveillance Systems that incorporate temperature and rainfall data to increase preparedness. I suggest highlighting the value of a multi-disease approach, preventing parallel single-disease systems or strategies, especially relevant for VBDs as many vector control strategies tackle multiple VBDs simultaneously (ie Integrated Vector Management, WHO recommended approach).

4. In line 126-127 “Vulnerability varies by population, with age, location, and urban setting influencing infection risk.” I suggest being more specific here. Which ages, locations, and urban settings are more at risk?

5. The authors may want to mention the new dengue vaccine, TAK-003, in the discussion, as a new potential government response, recommended by WHO

Reviewer #2: Summary and General Comments

Thank you for the opportunity to review this manuscript.

This manuscript addresses a topic of high relevance: the intersection of climate change, urbanization, and dengue transmission in LMICs. However, the study suffers from significant methodological flaws and data inconsistencies that prevent its publication in its current form.

I observed that the supplementary tables (S1 and S2) correctly list 65 studies. However, the final Reference list contains untreated duplicates (ref. 71 identical to 73; ref. 75 identical to 79; ref. 76 identical to 81) and software formatting errors (ref. 72).

Furthermore, I reinforce a serious inconsistency: the text claims that Brazil is one of the most represented countries, but there are no Brazilian studies listed in tables S1 and S2 or in the bibliography. This absence directly contradicts the statement that the country is one of the most represented. This suggests that either the supplementary data are incomplete, or the textual conclusion was based on studies that were not included in the final analysis. Please verify this.

I also have a concern regarding the title versus the content. The title highlights "Government Actions", but your results admit that only 29% of the studies documented this. It seems the paper promises more on government strategies than it actually delivers; the focus is heavily skewed towards climate (65%). Additionally, I question the choice of a "rapid review" for an endemic problem like Dengue. A full systematic review would have been more appropriate to provide the necessary depth.

Below are the errors I verified and my comments line by line:

- Line 8: Aedes aegypti must always be in italics.

- Line 85 and Abstract: In the phrase "though many were interfered by", the phrasing sounds strange; rewrite using another term like "hindered" which fits the context better.

- Line 94: "tropic" should be "tropical".

- Line 134 (Introduction): I felt the lack of a paragraph speaking about the role of temperature on the vector and dengue transmission. How does it affect it? Only the role of El Niño in Central America was mentioned, but that is all. You could report the regulatory and amplifying role that temperature has, that high and low temperatures naturally regulate cases, and that with the increase in global average temperatures, we could have both an increase in dengue occurrence regions and a decrease in hotter regions. Saying this is important as it links directly with the point of the article which is climate change and the increase in global temperatures. El Niño is important, but it is a climate modulator, not an effect of climate change.

- Lines 152 to 156: This entire paragraph is repeating from the "Study Design", please exclude it.

- Methods (Time Window): The study only covers 2020 to 2024. For a paper on climate change, a 4-year window seems extremely short. By doing this, you are discarding important longitudinal studies published before 2020 that are crucial for understanding these trends.

- Table 1, Language row: This may have limited the results quite a lot. Of the 65 articles selected in the end, much more could have been obtained if documents in Spanish and/or Portuguese had been included. You missed important regional databases like LILACS or SciELO. Excluding these introduces a severe geographical bias, as Latin American science on Dengue is often published in these languages.

- Line 215: Most studies are from South and Southeast Asia; as I mentioned, the limitation of the document being in English and the exclusion of regional databases may have limited the results a lot. Not including many studies from Lusophone and Hispanophone countries that are constantly in contact with dengue in Africa and the Americas.

- Line 223 on "Brazil": I checked the two tables mentioned so far and did not find any mention of Brazil. Revise.

- Lines 255 to 258: For this very reason, I believe a paragraph describing the possible effects of temperature should be included in the introduction.

- Line 356: Good that the language bias was mentioned, it really affected the representativeness of the data.

Besides this, I also observed duplicates in the list of references:

- Reference 71 identical to 73

- Reference 75 identical to 79

- Reference 76 identical to 81

**********

PLOS authors have the option to publish the peer review history of their article (what does this mean?). If published, this will include your full peer review and any attached files.

If you choose “no”, your identity will remain anonymous but your review may still be made public.

**Do you want your identity to be public for this peer review?**  For information about this choice, including consent withdrawal, please see our Privacy Policy.

Reviewer #1: **Yes:**  Frederike Kooiman

Reviewer #2: No

[NOTE: If reviewer comments were submitted as an attachment file, they will be attached to this email and accessible via the submission site. Please log into your account, locate the manuscript record, and check for the action link "View Attachments". If this link does not appear, there are no attachment files.]

**Figure resubmission:**

While revising your submission, we strongly recommend that you use PLOS’s NAAS tool (https://ngplosjournals.pagemajik.ai/artanalysis) to test your figure files. NAAS can convert your figure files to the TIFF file type and meet basic requirements (such as print size, resolution), or provide you with a report on issues that do not meet our requirements and that NAAS cannot fix.

After uploading your figures to PLOS’s NAAS tool - https://ngplosjournals.pagemajik.ai/artanalysis, NAAS will process the files provided and display the results in the "Uploaded Files" section of the page as the processing is complete. If the uploaded figures meet our requirements (or NAAS is able to fix the files to meet our requirements), the figure will be marked as "fixed" above. If NAAS is unable to fix the files, a red "failed" label will appear above. When NAAS has confirmed that the figure files meet our requirements, please download the file via the download option, and include these NAAS processed figure files when submitting your revised manuscript.

**Reproducibility:**

To enhance the reproducibility of your results, we recommend that authors of applicable studies deposit laboratory protocols in protocols.io, where a protocol can be assigned its own identifier (DOI) such that it can be cited independently in the future. Additionally, PLOS ONE offers an option to publish peer-reviewed clinical study protocols. Read more information on sharing protocols at https://plos.org/protocols?utm_medium=editorial-email&utm_source=authorletters&utm_campaign=protocols

# Author comment: Author response to Decision Letter 1

**DOI:** 10.1371/journal.pntd.0013758.r002

29 May 2026

Attachment Submitted filename: Responses to Reviewers (PNTD-D-25-02012).docx

# Peer review: Decision Letter 1

**DOI:** 10.1371/journal.pntd.0013758.r003

**Authors:** David Safronetz

15 Jun 2026

PNTD-D-25-02012R1

Battling Dengue in a Warming World: How Climate and Urbanization Shape Transmission in Low- and Middle-Income Countries (A Rapid Review)

PLOS Neglected Tropical Diseases

Dear Dr. Yu,

Thank you for submitting your manuscript to PLOS Neglected Tropical Diseases. After careful consideration, we feel that it has merit but does not fully meet PLOS Neglected Tropical Diseases's publication criteria as it currently stands. Therefore, we invite you to submit a revised version of the manuscript that addresses the points raised during the review process.

Please submit your revised manuscript by Jul 15 2026 11:59PM. If you will need more time than this to complete your revisions, please reply to this message or contact the journal office at plosntds@plos.org. When you're ready to submit your revision, log on to https://www.editorialmanager.com/pntd/ and select the 'Submissions Needing Revision' folder to locate your manuscript file.

Please include the following items when submitting your revised manuscript:

* A letter that responds to each point raised by the editor and reviewer(s). You should upload this letter as a separate file labeled 'Response to Reviewers'. This file does not need to include responses to any formatting updates and technical items listed in the 'Journal Requirements' section below.

* A marked-up copy of your manuscript that highlights changes made to the original version. You should upload this as a separate file labeled 'Revised Manuscript with Track Changes'.

* An unmarked version of your revised paper without tracked changes. You should upload this as a separate file labeled 'Manuscript'.

If you would like to make changes to your financial disclosure, competing interests statement, or data availability statement, please make these updates within the submission form at the time of resubmission. Guidelines for resubmitting your figure files are available below the reviewer comments at the end of this letter.

As the corresponding author, your ORCID iD is verified in the submission system and will appear in the published article. PLOS supports the use of ORCID, and we encourage all coauthors to register for an ORCID iD and use it as well. Please encourage your coauthors to verify their ORCID iD within the submission system before final acceptance, as unverified ORCID iDs will not appear in the published article. Only the individual author can complete the verification step; PLOS staff cannot verify ORCID iDs on behalf of authors.

We look forward to receiving your revised manuscript.

Kind regards,

David Safronetz, Ph.D.

Section Editor

PLOS Neglected Tropical Diseases

David Safronetz

Section Editor

PLOS Neglected Tropical Diseases

Shaden Kamhawi

co-Editor-in-Chief

PLOS Neglected Tropical Diseases

orcid.org/0000-0003-4304-636XX

Paul Brindley

co-Editor-in-Chief

PLOS Neglected Tropical Diseases

orcid.org/0000-0003-1765-0002

**Additional Editor Comments (if provided):**

**Journal Requirements:**

If the reviewer comments include a recommendation to cite specific previously published works, please review and evaluate these publications to determine whether they are relevant and should be cited. There is no requirement to cite these works unless the editor has indicated otherwise.

1) <carina-action-element class="ng-star-inserted" style="color: rgba(0, 0, 0, 0.87); font-family: sans-serif; font-size: 12px;">Please note that your Data Availability Statement is currently missing </carina-action-element><carina-action-element class="ng-star-inserted" style="color: rgba(0, 0, 0, 0.87); font-family: sans-serif; font-size: 12px;">the DOI/accession number of each dataset OR a direct link to access each dataset</carina-action-element><carina-action-element class="ng-star-inserted" style="color: rgba(0, 0, 0, 0.87); font-family: sans-serif; font-size: 12px;">. If your manuscript is accepted for publication, you will be asked to provide these details on a very short timeline. We therefore suggest that you provide this information now, though we will not hold up the peer review process if you are unable.</carina-action-element>

2) <carina-action-element class="ng-star-inserted">As required by our policy on Data Availability, please ensure your manuscript or supplementary information includes the following:</carina-action-element>

--><carina-action-element class="ng-star-inserted">- A numbered table of all studies identified in the literature search, including those that were excluded from the analyses.</carina-action-element>

<carina-action-element class="ng-star-inserted">- For every excluded study, the table should list the reason(s) for exclusion.</carina-action-element>

<carina-action-element class="ng-star-inserted">- If any of the included studies are unpublished, include a link (URL) to the primary source or detailed information about how the content can be accessed.</carina-action-element>

<carina-action-element class="ng-star-inserted">- A table of all data extracted from the primary research sources for the systematic review and/or meta-analysis. The table must include the following information for each study:</carina-action-element>

<carina-action-element class="ng-star-inserted">- Name of data extractors and date of data extraction</carina-action-element>

<carina-action-element class="ng-star-inserted">- Confirmation that the study was eligible to be included in the review.</carina-action-element>

<carina-action-element class="ng-star-inserted">- All data extracted from each study for the reported systematic review and/or meta-analysis that would be needed to replicate your analyses.</carina-action-element>

<carina-action-element class="ng-star-inserted">- If data or supporting information were obtained from another source (e.g. correspondence with the author of the original research article), please provide the source of data and dates on which the data/information were obtained by your research group.</carina-action-element>

<carina-action-element class="ng-star-inserted">- If applicable for your analysis, a table showing the completed risk of bias and quality/certainty assessments for each study or outcome. Please ensure this is provided for each domain or parameter assessed. For example, if you used the Cochrane risk-of-bias tool for randomized trials, provide answers to each of the signalling questions for each study. If you used GRADE to assess certainty of evidence, provide judgements about each of the quality of evidence factor. This should be provided for each outcome.</carina-action-element>

<carina-action-element class="ng-star-inserted">- An explanation of how missing data were handled.</carina-action-element>

<carina-action-element class="ng-star-inserted">This information can be included in the main text, supplementary information, or relevant data repository. Please note that providing these underlying data is a requirement for publication in this journal, and if these data are not provided your manuscript might be rejected.</carina-action-element>

<carina-action-element class="ng-star-inserted"></carina-action-element>

<carina-action-element class="ng-star-inserted"></carina-action-element>

**Reviewers' comments:**

Reviewer's Responses to Questions

**Key Review Criteria Required for Acceptance?**

As you describe the new analyses required for acceptance, please consider the following:

**Methods**

-Are the objectives of the study clearly articulated with a clear testable hypothesis stated?

-Is the study design appropriate to address the stated objectives?

-Is the population clearly described and appropriate for the hypothesis being tested?

-Is the sample size sufficient to ensure adequate power to address the hypothesis being tested?

-Were correct statistical analysis used to support conclusions?

-Are there concerns about ethical or regulatory requirements being met?

Reviewer #2: -Are the objectives of the study clearly articulated with a clear testable hypothesis stated?

yes

-Is the study design appropriate to address the stated objectives?

Yes, as they claim to be a rapid review

-Is the population clearly described and appropriate for the hypothesis being tested?

Yes

-Is the sample size sufficient to ensure adequate power to address the hypothesis being tested?

No, but the authors adequately address the limitation

-Were correct statistical analysis used to support conclusions?

Yes

-Are there concerns about ethical or regulatory requirements being met?

Yes

**********

**Results**

-Does the analysis presented match the analysis plan?

-Are the results clearly and completely presented?

-Are the figures (Tables, Images) of sufficient quality for clarity?

Reviewer #2: -Does the analysis presented match the analysis plan?

Yes

-Are the results clearly and completely presented?

Yes

-Are the figures (Tables, Images) of sufficient quality for clarity?

Yes

**********

**Conclusions**

-Are the conclusions supported by the data presented?

-Are the limitations of analysis clearly described?

-Do the authors discuss how these data can be helpful to advance our understanding of the topic under study?

-Is public health relevance addressed?

Reviewer #2: -Are the conclusions supported by the data presented?

Yes

-Are the limitations of analysis clearly described?

Yes

-Do the authors discuss how these data can be helpful to advance our understanding of the topic under study?

Yes

-Is public health relevance addressed?

Yes

**********

**Editorial and Data Presentation Modifications?**

Use this section for editorial suggestions as well as relatively minor modifications of existing data that would enhance clarity. If the only modifications needed are minor and/or editorial, you may wish to recommend “Minor Revision” or “Accept”.

Reviewer #2: (No Response)

**********

**Summary and General Comments**

Use this section to provide overall comments, discuss strengths/weaknesses of the study, novelty, significance, general execution and scholarship. You may also include additional comments for the author, including concerns about dual publication, research ethics, or publication ethics. If requesting major revision, please articulate the new experiments that are needed.

Reviewer #2: Thank you again for the opportunity to review this work. The authors have properly addressed the majority of my concerns, and the manuscript has been significantly improved. However, before I can fully endorse the publication, there are two minor, yet methodologically critical, discrepancies between the rebuttal letter and the revised manuscript that must be resolved:

1- SciELO and LILACS databases: In the "Response to Reviewers", the authors state they conducted an additional search in SciELO and LILACS via the Global Index Medicus to address regional bias. While I appreciate this effort, this supplementary search is not mentioned in the manuscript's Methods section and the PRISMA flow diagram. This additional search must be formally documented in the text, and the PRISMA flowchart must be updated to reflect the new numbers.

2- Justification for the 2020-2024 study period: The rebuttal letter provides a solid rationale for this specific 4-year window, explaining that it intentionally aligns with the "COVID-19 era" and its unique disruptions to health systems and vector control. However, this justification is entirely missing from the manuscript. A short timeframe for a climate change review requires explicit justification within the text so future readers understand the reasoning behind this methodological choice.

Once these two clarifications are formally incorporated into the manuscript text, I will be happy to endorse its publication.

**********

PLOS authors have the option to publish the peer review history of their article (what does this mean?). If published, this will include your full peer review and any attached files.

If you choose “no”, your identity will remain anonymous but your review may still be made public.

**Do you want your identity to be public for this peer review?**  For information about this choice, including consent withdrawal, please see our Privacy Policy.

Reviewer #2: **Yes:**  Iuri Valerio Graciano Borges

[NOTE: If reviewer comments were submitted as an attachment file, they will be attached to this email and accessible via the submission site. Please log into your account, locate the manuscript record, and check for the action link "View Attachments". If this link does not appear, there are no attachment files.]

**Figure resubmission:**

While revising your submission, we strongly recommend that you use PLOS’s NAAS tool (https://ngplosjournals.pagemajik.ai/artanalysis) to test your figure files. NAAS can convert your figure files to the TIFF file type and meet basic requirements (such as print size, resolution), or provide you with a report on issues that do not meet our requirements and that NAAS cannot fix.

After uploading your figures to PLOS’s NAAS tool - https://ngplosjournals.pagemajik.ai/artanalysis, NAAS will process the files provided and display the results in the "Uploaded Files" section of the page as the processing is complete. If the uploaded figures meet our requirements (or NAAS is able to fix the files to meet our requirements), the figure will be marked as "fixed" above. If NAAS is unable to fix the files, a red "failed" label will appear above. When NAAS has confirmed that the figure files meet our requirements, please download the file via the download option, and include these NAAS processed figure files when submitting your revised manuscript.

**Reproducibility:**

To enhance the reproducibility of your results, we recommend that authors of applicable studies deposit laboratory protocols in protocols.io, where a protocol can be assigned its own identifier (DOI) such that it can be cited independently in the future. Additionally, PLOS ONE offers an option to publish peer-reviewed clinical study protocols. Read more information on sharing protocols at https://plos.org/protocols?utm_medium=editorial-email&utm_source=authorletters&utm_campaign=protocols

# Author comment: Author response to Decision Letter 2

**DOI:** 10.1371/journal.pntd.0013758.r004

1 Jul 2026

Attachment Submitted filename: Rebuttal Letter (PNTD-D-25-02012R2).docx

# Editor report: Decision Letter 2

**DOI:** 10.1371/journal.pntd.0013758.r005

**Authors:** David Safronetz

3 Jul 2026

Dear Dr. Yu,

We are pleased to inform you that your manuscript 'Battling Dengue in a Warming World: How Climate and Urbanization Shape Transmission in Low- and Middle-Income Countries (A Rapid Review)' has been provisionally accepted for publication in PLOS Neglected Tropical Diseases.

Before your manuscript can be formally accepted you will need to complete some formatting changes, which you will receive in a follow up email. A member of our team will be in touch with a set of requests.

Please note that your manuscript will not be scheduled for publication until you have made the required changes, so a swift response is appreciated.

IMPORTANT: The editorial review process is now complete. PLOS will only permit corrections to spelling, formatting or significant scientific errors from this point onwards. Requests for major changes, or any which affect the scientific understanding of your work, will cause delays to the publication date of your manuscript.

Should you, your institution's press office or the journal office choose to press release your paper, you will automatically be opted out of early publication. We ask that you notify us now if you or your institution is planning to press release the article. All press must be co-ordinated with PLOS.

Thank you again for supporting Open Access publishing; we are looking forward to publishing your work in PLOS Neglected Tropical Diseases.

Best regards,

David Safronetz, Ph.D.

Section Editor

PLOS Neglected Tropical Diseases

David Safronetz

Section Editor

PLOS Neglected Tropical Diseases

Shaden Kamhawi

co-Editor-in-Chief

PLOS Neglected Tropical Diseases

orcid.org/0000-0003-4304-636XX

Paul Brindley

co-Editor-in-Chief

PLOS Neglected Tropical Diseases

orcid.org/0000-0003-1765-0002

***********************************************************

p.p1 {margin: 0.0px 0.0px 0.0px 0.0px; line-height: 16.0px; font: 14.0px Arial; color: #323333; -webkit-text-stroke: #323333}span.s1 {font-kerning: none

# Editor report: Acceptance letter

**DOI:** 10.1371/journal.pntd.0013758.r006

**Authors:** David Safronetz

Dear Dr. Yu,

We are delighted to inform you that your manuscript, "Battling Dengue in a Warming World: How Climate and Urbanization Shape Transmission in Low- and Middle-Income Countries (A Rapid Review)," has been formally accepted for publication in PLOS Neglected Tropical Diseases.

We have now passed your article onto the PLOS Production Department who will complete the rest of the publication process. All authors will receive a confirmation email upon publication.

The corresponding author will soon be receiving a typeset proof for review, to ensure errors have not been introduced during production. Please review the PDF proof of your manuscript carefully, as this is the last chance to correct any scientific or type-setting errors. Please note that major changes, or those which affect the scientific understanding of the work, will likely cause delays to the publication date of your manuscript. Note: Proofs for Front Matter articles (Editorial, Viewpoint, Symposium, Review, etc...) are generated on a different schedule and may not be made available as quickly.

Soon after your final files are uploaded, the early version of your manuscript will be published online unless you opted out of this process. The date of the early version will be your article's publication date. The final article will be published to the same URL, and all versions of the paper will be accessible to readers.

For Research Articles, you will receive an invoice from PLOS for your publication fee after your manuscript has reached the completed accept phase. If you receive an email requesting payment before acceptance or for any other service, this may be a phishing scheme. Learn how to identify phishing emails and protect your accounts at https://explore.plos.org/phishing.

Thank you again for supporting open-access publishing; we are looking forward to publishing your work in PLOS Neglected Tropical Diseases.

Best regards,

Shaden Kamhawi

co-Editor-in-Chief

PLOS Neglected Tropical Diseases

Paul Brindley

co-Editor-in-Chief

PLOS Neglected Tropical Diseases
